# A Polarised Population of Dynamic Microtubules Mediates Homeostatic Length Control in Animal Cells

**DOI:** 10.1371/journal.pbio.1000542

**Published:** 2010-11-16

**Authors:** Remigio Picone, Xiaoyun Ren, Kenzo D. Ivanovitch, Jon D. W. Clarke, Rachel A. McKendry, Buzz Baum

**Affiliations:** 1 Centre for Mathematics and Physics in the Life Sciences and Experimental Biology, University College London, London, United Kingdom; 2 London Centre for Nanotechnology, London, United Kingdom; 3 Department of Medicine, University College London, London, United Kingdom; 4 Medical Research Council Laboratory for Molecular Cell Biology, University College London, London, United Kingdom; 5 Medical Research Council Centre for Developmental Neurobiology, King's College London, London, United Kingdom; University of California, Davis, United States of America

## Abstract

Because physical form and function are intimately linked, mechanisms that maintain cell shape and size within strict limits are likely to be important for a wide variety of biological processes. However, while intrinsic controls have been found to contribute to the relatively well-defined shape of bacteria and yeast cells, the extent to which individual cells from a multicellular animal control their plastic form remains unclear. Here, using micropatterned lines to limit cell extension to one dimension, we show that cells spread to a characteristic steady-state length that is independent of cell size, pattern width, and cortical actin. Instead, homeostatic length control on lines depends on a population of dynamic microtubules that lead during cell extension, and that are aligned along the long cell axis as the result of interactions of microtubule plus ends with the lateral cell cortex. Similarly, during the development of the zebrafish neural tube, elongated neuroepithelial cells maintain a relatively well-defined length that is independent of cell size but dependent upon oriented microtubules. A simple, quantitative model of cellular extension driven by microtubules recapitulates cell elongation on lines, the steady-state distribution of microtubules, and cell length homeostasis, and predicts the effects of microtubule inhibitors on cell length. Together this experimental and theoretical analysis suggests that microtubule dynamics impose unexpected limits on cell geometry that enable cells to regulate their length. Since cells are the building blocks and architects of tissue morphogenesis, such intrinsically defined limits may be important for development and homeostasis in multicellular organisms.

## Abstract 2

An analysis of cells grown on micro-patterned lines, and of cells during zebrafish development, identifies a population of microtubules that align along the long axis of cells to mediate homeostatic length control.

## Author Summary

Because many physical processes change with scale, size control is a fundamental problem for living systems. While in some instances the size of a structure is directly determined by the dimensions of its individual constituents, many biological structures are dynamic, self-organising assemblies of relatively small component parts. How such assemblies are maintained within defined size limits remains poorly understood. Here, by confining cells to spread on lines, we show that animal cells reach a defined length that is independent of their volume and width. In searching for a “ruler” that might determine this axial limit to cell spreading, we identified a population of dynamic microtubule polymers that become oriented along the long axis of cells. This growing population of oriented microtubules drives extension of the spreading cell margin while, conversely, interactions with the cell margin promote microtubule depolymerisation, leading to cell shortening. Using a mathematical model we show that this coupling of dynamic microtubule polymerisation and depolymerisation with directed cell elongation is sufficient to explain the limit to cell spreading and cell length homeostasis. Because microtubules appear to regulate cell length in a similar way in the developing zebrafish neural tube, we suggest that this microtubule-dependent mechanism is likely to be of widespread importance for the regulation of cell and tissue geometry.

## Introduction

The physical properties of a system depend to a large extent upon its scale. Therefore, it is not surprising to find that many biological structures are maintained within relatively tightly constrained size limits [1],[2]. In some cases, the dimensions of macromolecular assemblies are enforced by “molecular rulers” like titin, which helps to govern the length of the sarcomeric repeats in muscle [3]. However, many seemingly stable structures, such as metaphase spindles [4] and cilia [1], exist in a state of dynamic equilibrium in which a stable form arises from the collective action of a large number of molecular machines functioning in concert. Although mechanisms have been proposed for the control of the length of such polymers [1], through for example length-dependent microtubule depolymerisation [5], little is known about this fundamental and widespread biological phenomenon.

For unicellular organisms, intrinsic mechanisms have been identified that regulate cell shape [2],[6], maintain a steady-state cell size, and couple cell length and size [7]. However, it remains unclear whether similar controls regulate the dimensions of cells from multicellular animals, which, by virtue of not having a cell wall, assume a form that is plastic and a variable size, both of which depend to a large degree upon the extracellular tissue environment in which cells find themselves [8],[9]. Nevertheless, since form and function are intimately linked and vary from cell type to cell type, it seems likely that the shape of many animal cells will be maintained within intrinsically defined limits. Such behaviour has been observed in assays of cell spreading [10] and cell migration on planar adhesive substrates [11],[12]. Moreover, studies of cells on grooved, scratched, or patterned substrates have in some cases [13],[14] revealed limits to cell extension. In addition, regulated changes in cell geometry have long been known to drive a variety of morphogenesis movements in developing animals. During *Drosophila* development, for example, changes in epithelial cell shape and height are thought to drive internalisation of the ventral furrow [15]. Similarly, during neural tube development in zebrafish, individual neuroepithelial cells act together to form a double-layered epithelium with a defined width, even under conditions in which the entire structure is mis-positioned or duplicated [16].

Here, to systematically investigate the mechanisms that control animal cell geometry, we employed micro-contact printing [17] to generate adhesive lines of extracellular matrix that limit cell width but leave cells free to regulate their geometry in the other two dimensions (length and height). This analysis of cells spreading on adhesive lines reveals that animal cells can spread to a characteristic steady-state length that is independent of cell size, pattern width, and cortical actin, but is dependent on a population of microtubules that aligns along the long axis of cells as the result of interactions between microtubule plus ends and the cell cortex. Similarly, a population of oriented microtubules mediates length control in epithelial cells of the developing zebrafish neural tube. A mathematical model shows that cell length homeostasis in these cases can be quantitatively explained by the collective action of dynamic, orientated microtubules as they drive cell extension and undergo cortex-dependent catastrophe. Together, this experimental and theoretical analysis reveals a role for microtubules in homeostatic cell length control in HeLa cells, *Drosophila* S2R+ cells, and zebrafish neuroepithelial cells, suggesting that it may be an important general feature of animal cell biology.

## Results

### Cells on Micropatterned Lines Spread to a Steady State Length That Is Independent of Pattern Width and Cell Size

**Figure 1:**
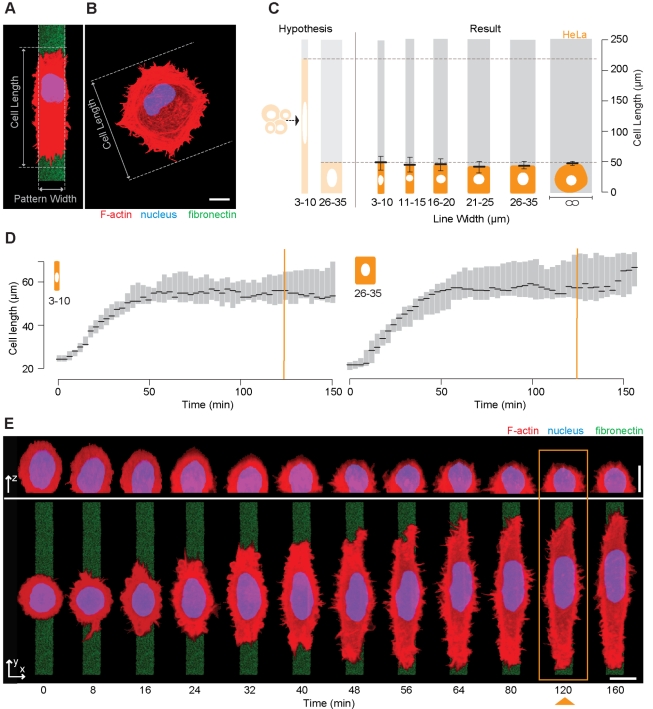
Length homeostasis in HeLa cells on patterned fibronectin lines. (A) HeLa cells on patterned 13-µm-wide fibronectin-FITC lines (green) were fixed and stained with TRITC-phalloidin to label actin filaments (red) and DAPI to label DNA (blue). Images depict *xz* and *xy* projections. The length of HeLa cells on patterned lines was defined as the distance, parallel to the patterned line, between distal-most cell extensions. (B) The length of cells on non-patterned fibronectin surfaces was defined as the greatest distance between any two points along the cell boundary (Feret's diameter). (C) Lengths of Hela cells were measured yielding an average length of 44±10 µm (*n* = 759) on patterned lines and 44±4 µm (*n* = 603) on non-patterned surfaces (∞). Dotted line indicates the lengths predicted for a constant cell height. (D and E) Hela cells spreading on lines of widths 3–10 µm and 25–35 µm were tracked and cell length semi-automatically calculated. During the first 60 min, the spreading velocity was 0.6±0.5 µm/min (*n* = 18) and 0.5±0.5 µm/min (*n* = 13) for narrow and wide patterned lines, respectively. Cells reached a steady-state length after ∼60 min. Orange line (D) and rectangle (E) indicate standard 120-min observation point as used in (C). Scale bars = 10 µm. Error bars denote upper and lower box plot quartiles.

To explore the intrinsic regulation of cell shape, we began by seeding a population of freshly harvested exponentially growing HeLa cells onto micropatterned fibronectin lines (Figure 1A) ranging in width from 3 to 35 µm, separated by non-adhesive polyethylene glycol, and onto equivalent non-patterned areas of the substrate (Figure 1B). Other researchers previously performed similar experiments by plating cells on grooved or scratched substrates [18]–[20] or on adhesive strips [13]. In our case, after allowing 2 h for cells to adhere to and spread on the micropatterned substrate, cell length was monitored using semi-automated software designed to remove user bias and to facilitate the analysis of large datasets (Figure S1). Cell length for this analysis was defined as the maximum distance, parallel to the patterned line, separating extensions at distal cell tips (Figure 1A). Unexpectedly, cells from an exponentially growing population (with a wide range of masses) spread to a relatively well-defined average length of 44±10 µm, which proved largely independent of line width and similar to that of cells on non-patterned substrates (∞) (Figure 1C). Cell spreading in this assay was accompanied by a corresponding change in cell height, as expected if cell volume is conserved (Figure 1E and data not shown). These observations were similar to those previously reported for fibroblasts on scratched substrates [13]. Because a fixed-time-point assay was used for this analysis, however, the independence of cell length and pattern width could be the result of either a constant rate of cell spreading or the action of a cell length control mechanism. When live-cell imaging was used to examine the kinetics of cell spreading, we observed HeLa cells spreading monotonically over a period of approximately 60 min, reaching a steady-state length that was independent of pattern width (Figure 1D and 1E). These data suggest that HeLa cells have an intrinsically defined length.

**Figure 2 pbio-1000542-g002:**
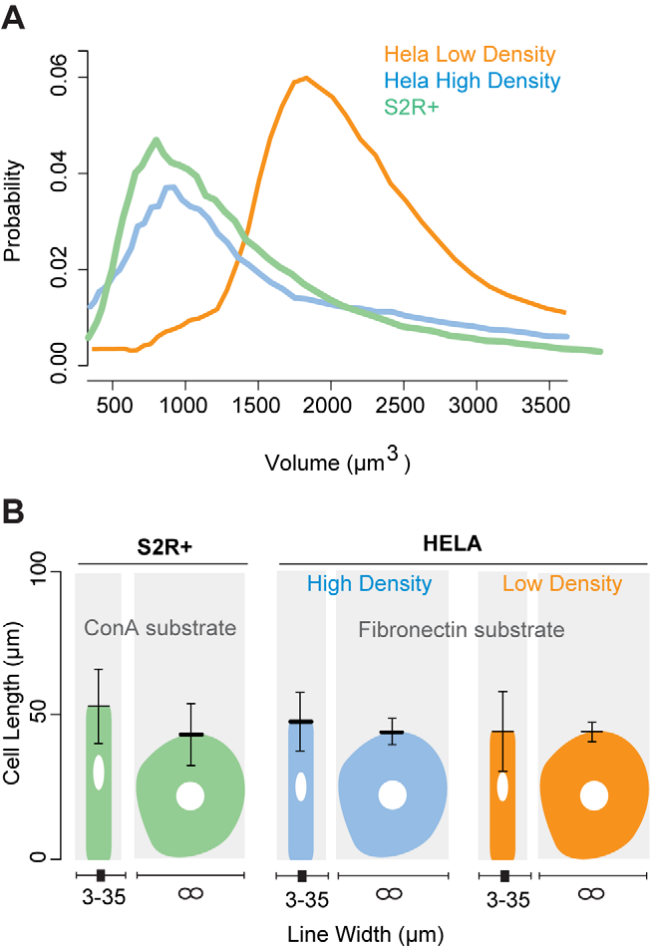
Cell length is not dependent on cell width, volume, or substrate type. (A) The volumes of S2R+ cells (green) and HeLa cells grown to 50% (orange, used in Figure 1) and 100% confluence (blue) were measured using a Coulter Counter. (B) Cells from these three cell populations were plated onto adhesive lines (ConA for S2R+ cells and fibronectin for HeLa cells) 3–35 µm in width and onto equivalent non-patterned surfaces (∞), and cell lengths were quantified. Error bars denote upper and lower box plot quartiles.

To determine whether cell length control is a peculiarity of HeLa cells spreading on a fibronectin substrate or a more general feature of animal cell biology, we repeated these experiments using the adherent *Drosophila* hemocyte-derived S2R+ cells [21], which are significantly smaller than HeLa cells (with a mean volume, measured using an automated cell counter, of 1,177±64 µm^3^ for *Drosophila* S2R+ cells compared to 2,121±1,116 µm^3^ for HeLa cells; Figure 2A). These cells do not adhere well to fibronection-coated substrates, but could be induced to spread on glass dishes coated with Concanavalin A (ConA) [15]. Once spreading was complete (5 h after plating), the length of these cells was measured. Like HeLa cells, patterned S2R+ cells were found to achieve a reproducible steady-state length that was relatively independent of their width (Figure 2B and data not shown). Moreover, despite their very different average volumes, S2R+ and HeLa cells had comparable resting lengths (55±9 µm and 44±10 µm, respectively). This observation suggested the possibility that the limit of an animal cell's long axis might be regulated independently of its volume. As a direct test of this hypothesis we altered the culturing conditions to obtain a population of HeLa cells grown to confluence that had an average volume of 1,233±835 µm^3^ (about half that of HeLa cells grown to 50% confluence; Figure 2A). When the two populations of differently sized HeLa cells were plated onto micro-contact-printed fibronectin lines of varying widths for 2 h, they were found to spread to statistically similar lengths (Figure 2B) despite having different heights (data not shown), confirming the independence of cell length and volume.

### Cell Length Control Is Dependent on a Population of Oriented Microtubules

**Figure 3:**
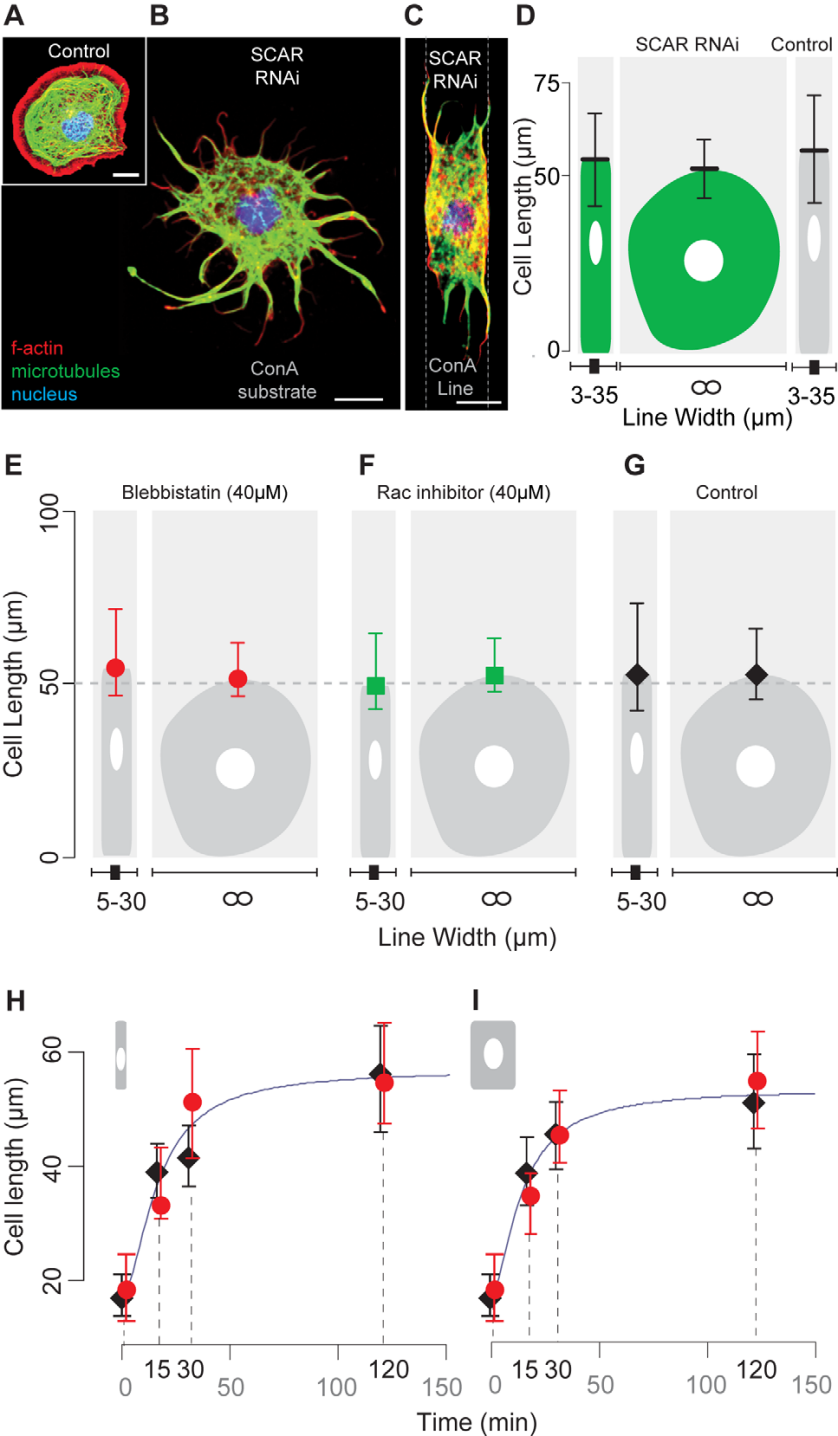
The extension limit of *Drosophila* cells is independent of lamellipodial actin. (A–C) S2R+ cells treated for 5 d with LacZ (control) or SCAR dsRNA were plated onto ConA lines and non-patterned ConA substrates (A). Depletion of SCAR produces spiky cells that lack all lamellipodia. Images show (A) a LacZ RNAi cell fixed and stained for actin filaments (red) and microtubules (green), and an equivalent SCAR RNAi cell on a non-patterned substrate (B) and a patterned ConA line (C). (D) The average lengths of SCAR RNAi S2R+ cells on patterned lines and on a non-patterned surface (∞) were calculated as 55±13 µm (*n* = 43) and 52±8 µm (*n* = 23), respectively. The equivalent measurement for LacZ RNAi cells was 58±15 µm (*n* = 66). (E–G) Lengths of HeLa cells treated with blebbistatin (40 µM) (E), NSC23766 (Rac inhibitor) (40 µM) (F), or DMSO (G) on fibronectin patterned lines and a non-patterned surface. (H and I) Plots show changes in the length of HeLa cells (determined after fixation) during a time course following treatment with blebbistatin (40 µM) (red circles) or DMSO (black diamonds) on thin ([H], 3 to 15 µm) or thick ([I], 16 to 35 µm) patterned fibronectin lines. Scale bar = 10 µm. Error bars denote upper and lower box plot quartiles.

It seemed unlikely that cell length control depends on plasma membrane tension, since dramatic changes in cell volume and line width had little impact on the length of HeLa or S2R+ cells, or on the spreading kinetics of HeLa cells on narrow and thick patterned lines (Figure 1D). In searching for mechanisms underlying cell length control we therefore turned to examine a possible role for the cytoskeleton [22], since actin filaments and microtubules have been implicated in the control of fixed-length structures [1],[3],[23] and in the regulation of cell length [6],[13] and cell spreading [18],[24]–[27]. We began by analysing the cytoskeletal changes that accompany cell spreading on micropatterned lines. Following attachment to a patterned surface and a brief period of blebbing (Figure 1E), cells developed spreading lamellipodia that quickly reached the edges of the patterned lines. While lamellipodia reaching laterally outside of the pattern into regions passified with polyethylene glycol underwent periodic cycles of extension and retraction [28], lamellipodia extending in the direction of cell elongation remained tightly bound to the adhesive substrate (data not shown). To test whether these actin-based lamellipodia play a role in cell elongation or cell length control, we took advantage of the efficacy of RNA interference (RNAi)–mediated gene silencing in fly cell culture to target SCAR/WAVE (hereafter SCAR) to selectively remove these structures from spreading cells [29]. Five days after treatment with control (LacZ) or SCAR double-stranded RNA (dsRNA) [30],[31], *Drosophila* S2R+ cells were seeded onto non-patterned substrates (Figure 3A and 3B) and onto micropatterned ConA lines (Figure 3C). Cell length was then assayed 5 h later (Figure 3D). As Figure 3C shows, SCAR RNAi cells assumed their typical RNAi phenotype, in which lamellipodia are replaced by long, radial, microtubule-rich processes [31]. Despite this, the majority of microtubule-based protrusions became oriented along the ConA lines. When we measured the distance parallel to the pattern between distal protrusion tips in these cells, we found that the length of SCAR RNAi cells was statistically indistinguishable from that of control cells (Figure 3D). Similarly, control RNAi and SCAR RNAi cells reached similar steady-state spread diameters on non-patterned substrates [31] (Figure 3D). Thus, although SCAR is required for the formation of lamellipodial-based protrusions, lamellipodial actin is not required for cell elongation and does not alter the steady-state length of microtubule-rich extensions. We then repeated these experiments in HeLa cells, using a Rac inhibitor [32] to compromise lamellipodial formation (Figure 3F). As with S2R+ cells, this did not alter cell length (compare Figure 3F and 3G). To test a possible role for actin-myosin-mediated cortical tension in the regulation of cell length, we also carried out a similar analysis using blebbistatin to inhibit Myosin II [33]. Once again, this did not affect the rate of cell spreading (Figure 3H and 3I) or resting cell length (54.3 [−8, +17] µm for HeLa cells [*n* = 345] on fibronectin lines, and 50.9 [−4, +11] µm for HeLa cells [*n* = 52] on non-patterned fibronectin) (Figure 3E). Although surprising, these data concur with previously reported work showing that the final spread area of fibroblasts and HeLa cells is relatively independent of membrane tension [34] and cortical actin [10].

**Figure 4:**
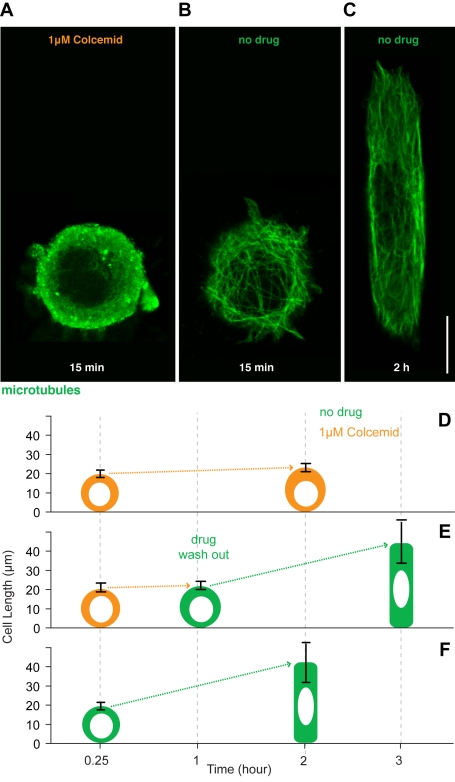
Microtubules lead elongation of HeLa cells on patterned line. Colcemid (1 µM) depolymerises microtubules ([A], compare with [B]) and stops cell elongation on fibronectin patterned lines (23±2 µm, *n* = 12) (D). After drug wash out microtubules re-grow and cells elongate (C), reaching a characteristic length of 42±11 µm (*n* = 38) (E). (F) shows average lengths of cells in DMSO solution at 15 min (20±2 µm, *n* = 15) and 2 h (43±10 µm, *n* = 35). In (D–F) average length is measured for DMSO- or colcemid-treated cells fixed at various times (15 min, 1 h, 2 h, and 3 h) after attachment to patterned lines. Scale bar = 10 µm. Error bars denote upper and lower box plot quartiles.

**Figure 5:**
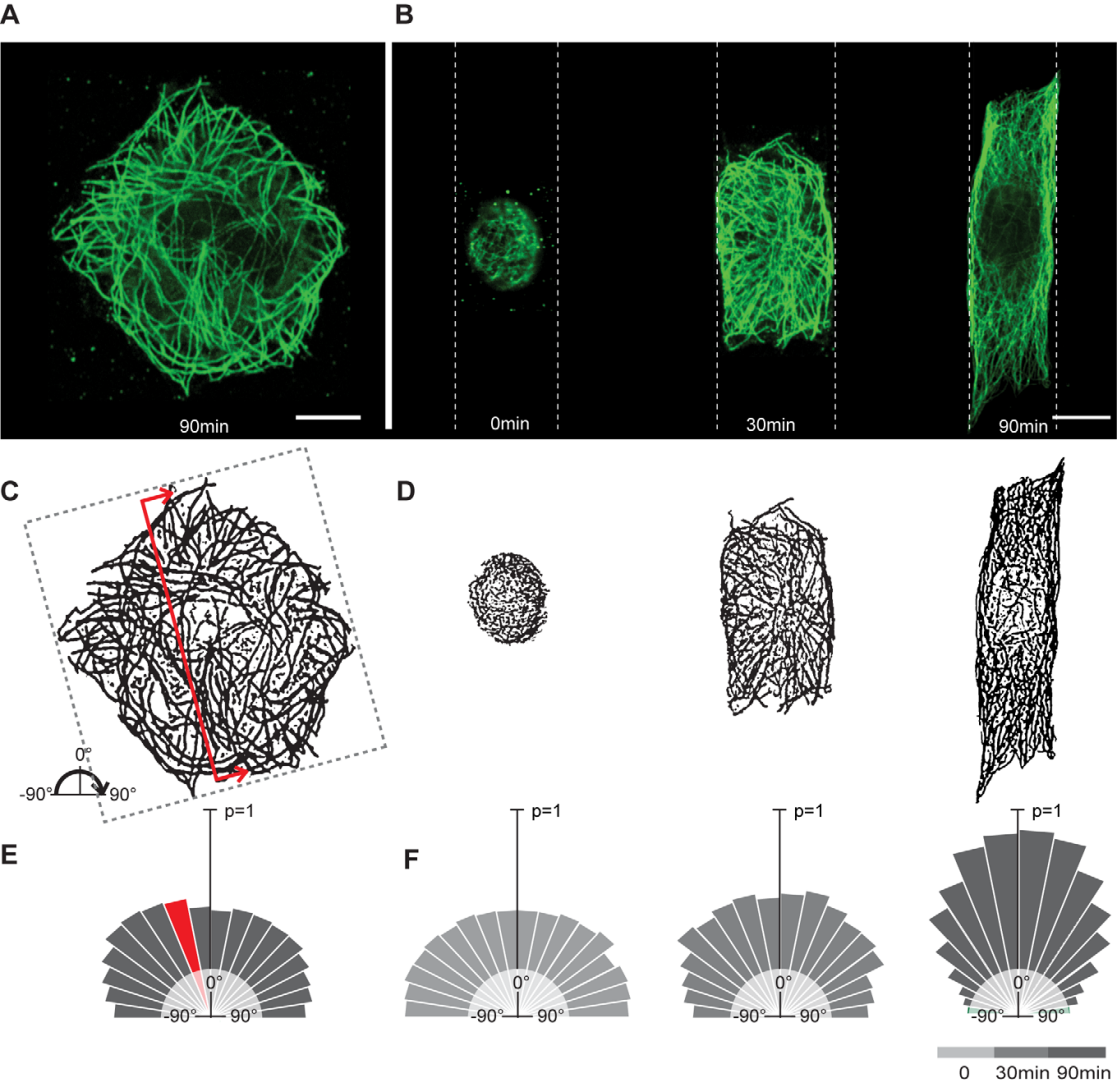
Microtubules become polarised during cell elongation and control cell length homeostasis. HeLa cells spreading on a non-patterned (A) or patterned (B) fibronectin substrate were fixed and microtubules labelled (green) 0, 30, and 90 min after attachment. Binary images of microtubules (C and D) were automatically processed (E and F) to reveal the extent of microtubule polarisation. This is depicted as an angular probability distribution for straight microtubules in non-patterned cells (*n* = 4) and in patterned cells at 0 min (*n* = 1), 30 min (*n* = 5), and 90 min (*n* = 4) after attachment. Microtubule polymer orientation was determined by normalising the amount of linear tubulin polymer at each angle by the total amount of linear polymer in the cell. Microtubules were identified based on overlap with straight lines (red line in [C]).

**Figure 6:**
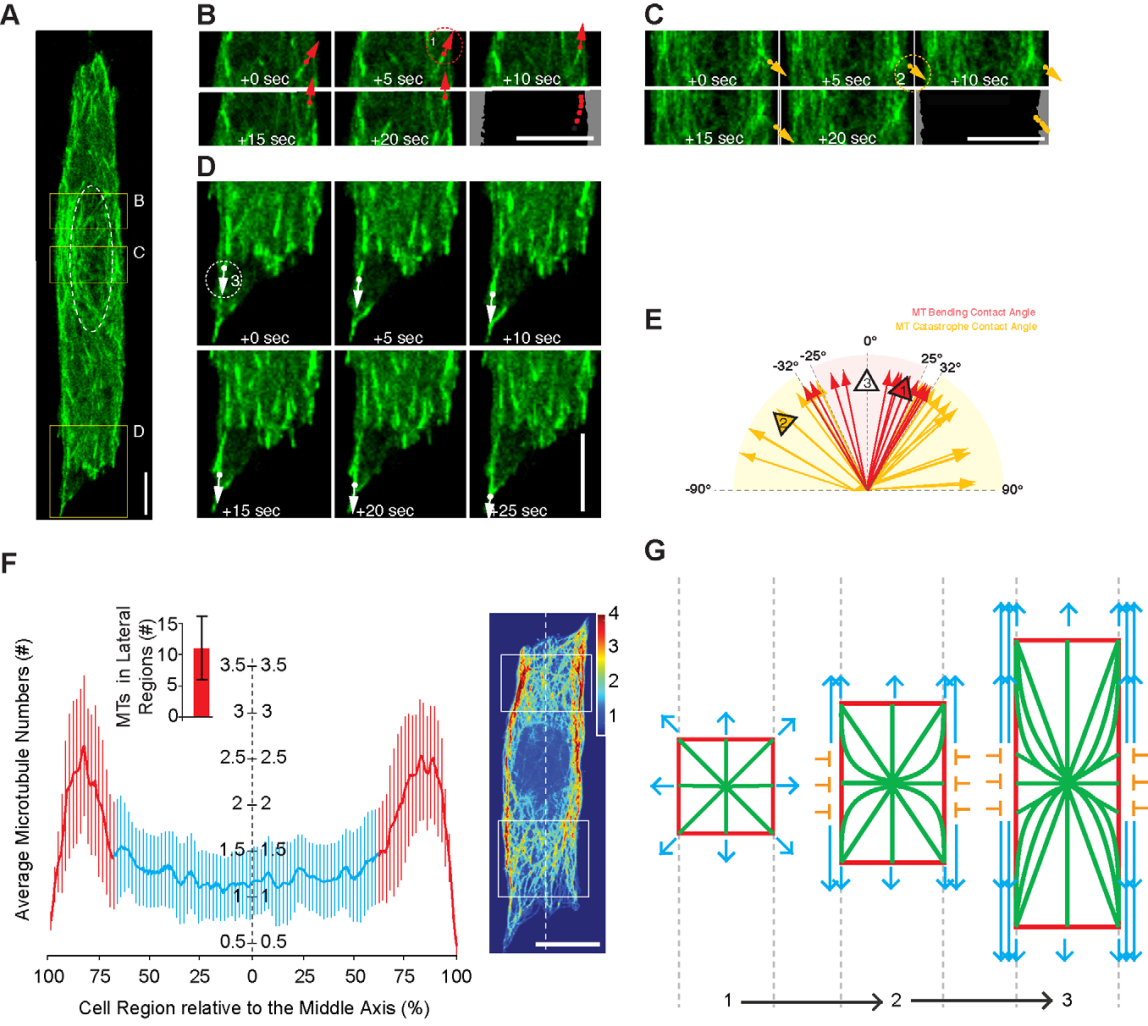
The fate of growing microtubules, to bend or to undergo catastrophe, depends on the contact angle with which they meet the cell cortex. (A–D) EB3-GFP-labelled microtubule plus ends were tracked in the perinuclear region of an HeLa cell, close to the site at which the majority of microtubules are nucleated ([A], nucleus labelled by white dotted line). Growing EB3-GFP-labelled microtubules that interact with the cell cortex (dotted circles) either bend to change direction ([B], red arrowhead, EB3-GFP spot 1) or undergo catastrophe ([C], yellow arrowhead, EB3-GFP spot 2). Through this process, a majority of microtubule plus ends become oriented parallel to the cell long axis and move towards the cell ends ([D], white arrowhead, EB3-GFP spot 3). (E) The fate of microtubules contacting the cell cortex depends on the contact angle (*n* = 39). An angle of between −25° and 25° (red) leads to a change in direction, whereas an angle of between 32° and 90° (or between −90° and −32°, yellow), leads to catastrophe. For microtubules with contact angles between 25° and 32° (or −32° and −25°), bending or catastrophe events appear to occur with similar probability. The black arrowheads in (E) refer to the EB3-GFP-labelled microtubules marked in (B–D). Quantitatively similar results were seen for EB1-GFP-labelled microtubule plus ends in S2 cells (see Video S3). (F) Microtubule (MT) density is shown plotted against cell width for HeLa cells on pattern lines at steady state (like that shown on right). Microtubule intensities were measured in cells (*n* = 20) labelled with FITC-conjugated anti-α-tubulin antibodies within regions just distal to the nucleus (examples marked by the rectangles in the right-hand image). Average microtubule numbers were then determined by normalising the intensity signal based upon the fluorescence of single microtubules. The histogram insert shows average number of microtubules (11±5) aligned within ∼30% of the cell edge along the lateral edge of cells (marked in red in large graph). These regions lead during cell elongation (see cell on right). (G) Diagram showing polarisation of microtubules (green lines) and the cell cortex (red rectangle) as the result of interactions between microtubules and the lateral cell cortex. Depending on the angle of incidence, contact induces microtubules to re-orient along the long cell axis (blue arrow heads) or to undergo catastrophe (orange Ts). Scale bar = 5 µm. Error bars denote the standard deviation.

Having failed to identify a role for the actin cytoskeleton, these data prompted us to examine the function of microtubules, which have previously been implicated in cell spreading [20],[24]–[26],[35] and in the generation of an elongated cell shape [36],[37]. Strikingly, in the presence of the microtubule inhibitor colcemid [38], HeLa cells were unable to elongate (Figure 4A and 4D). This effect was reversible since cells re-spread to their characteristic length after the drug was washed out and the microtubule cytoskeleton re-established (Figure 4C, 4E, and 4F). Cell spreading in this system therefore requires microtubules. To gain mechanistic insight into their precise role, we then analysed microtubule organisation at intervals during cell spreading (Figure 5). Cell elongation was accompanied by a progressive polarisation of the microtubule cytoskeleton, as microtubules concentrated on the basal part of the cell (Figure S3) became aligned along the long cell axis (Figure 5B and 5D; quantified in Figure 5F). Strikingly, a similar re-alignment was observed over shorter time scales as individual growing microtubules in cells at steady state became oriented to lie along the long cell axis (Figure 6A), as the result of contacts between growing microtubule plus tips (marked with EB3-GFP) and the ruffling cell margin at the interface between an adhesive and non-adhesive substrate (Figure 6B and 6C). This cortex-induced change in microtubule direction was similar to that previously described in yeast [6], animal cells [20],[39], and plant cells [40].

Interestingly, the fate of each growing microtubule meeting the cortex depended strongly on the angle of contact (Figure 6B, 6C, and 6E), such that microtubule plus ends contacting the cortex at a steep angle (32°–90° from the long line axis) underwent catastrophe, while others contacting the cortex at less of an angle (0°–25°) changed their direction of growth to run parallel to the cell edge. This explains the low probability of finding microtubules aligned at an angle of between 30° and 90° with respect to the long cell axis in Figure 5 (or −30° and −90° according to the notation in Figure 6C). As a result of this angular dependency, the microtubule cytoskeleton reached a highly polarised equilibrium state, with the majority of microtubules running along the cell edge (Figure 6A; quantified in Figure 6F; explained in Figure 6G). Importantly, this angular dependency of microtubule catastrophe/bending resulted in overt polarisation of the microtubule cytoskeleton. We also observed oriented microtubules leading during cell extension (Figure 6D and 6F; Video S1, S2, S3). Taken together these data suggest that microtubules oriented parallel to the adhesive boundary are strong candidates for drivers of cell spreading and the source of intrinsic cell length control.

### A Simple Model of Microtubule-Based Cell Spreading Recapitulates Cell Length Control

**Figure 7:**
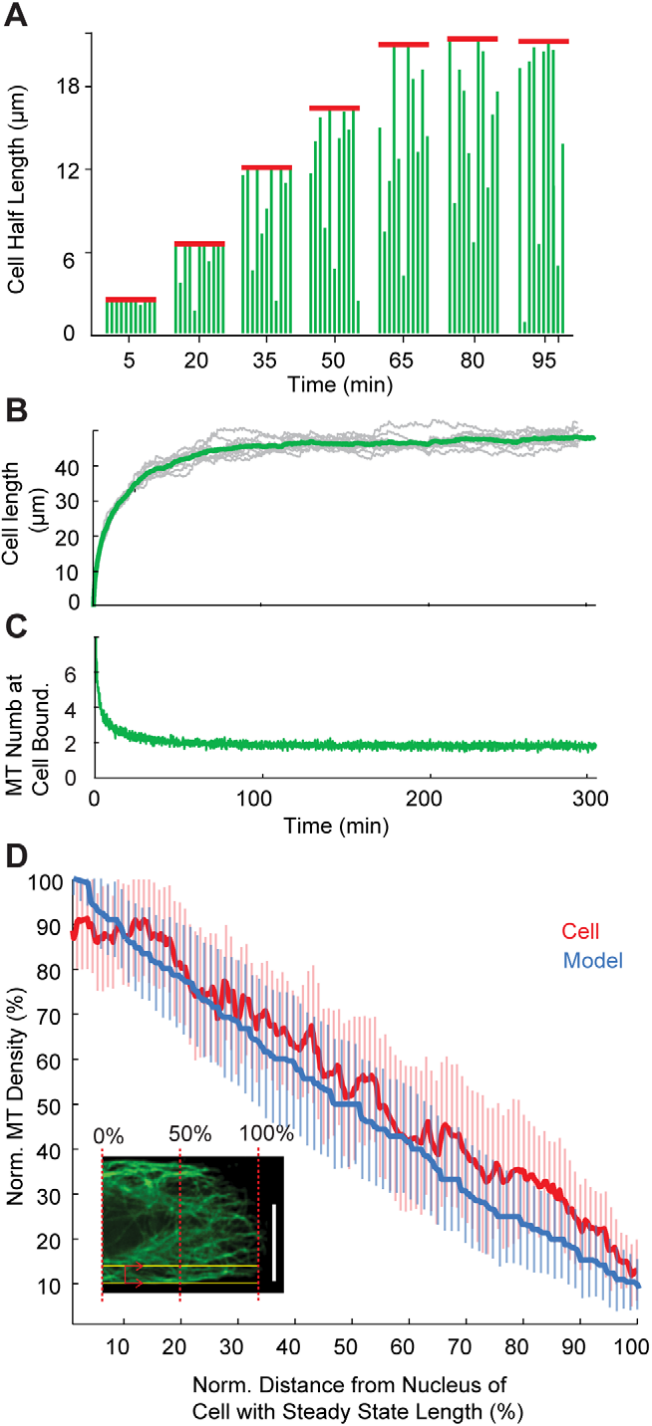
A model constructed based upon the interaction between parallel microtubules and a free cell edge. See Materials and Methods for more details. (A) Simulation of cell extension for *N*_m_ = 11 and α = 8 (other model parameters as shown in Table 1). The model was then used to explore the role of microtubule (green lines) number (*N*_m_) and the extent of microtubule cooperation (α) in cell elongation (red line) and cell length control. (B) Cell elongation kinetics observed in the model using α = 8 and *N*_m_ = 11 (other model parameters as in Table 1) recapitulates that seen in experiments. Stochastic simulations of cell elongation kinetics are shown in gray and average values in green. (C) Numbers of microtubule (MT) plus ends contacting the cell end during cell elongation in simulations using α = 8 and *N*_m_ = 11 (other model parameters as in Table 1). (D) Changes in microtubule density were plotted along the long cell axis for cells in the model (blue line) and in experiment (red line) at steady state. Changes in experimentally determined microtubule fluorescence and microtubule density obtained from simulations are shown as percentages of cell length, so that values across cells could be grouped to assess average cell behaviour. (Inset) To calculate the microtubule density across the edge of a HeLa cell on a patterned line, we measured microtubule (green) fluorescence intensity over the half cell length (scanning line in red) within 30% of the cell margin (i.e., within the yellow lines). Scale bar = 10 µm. Error bars denote the standard deviation.

**Figure 8:**
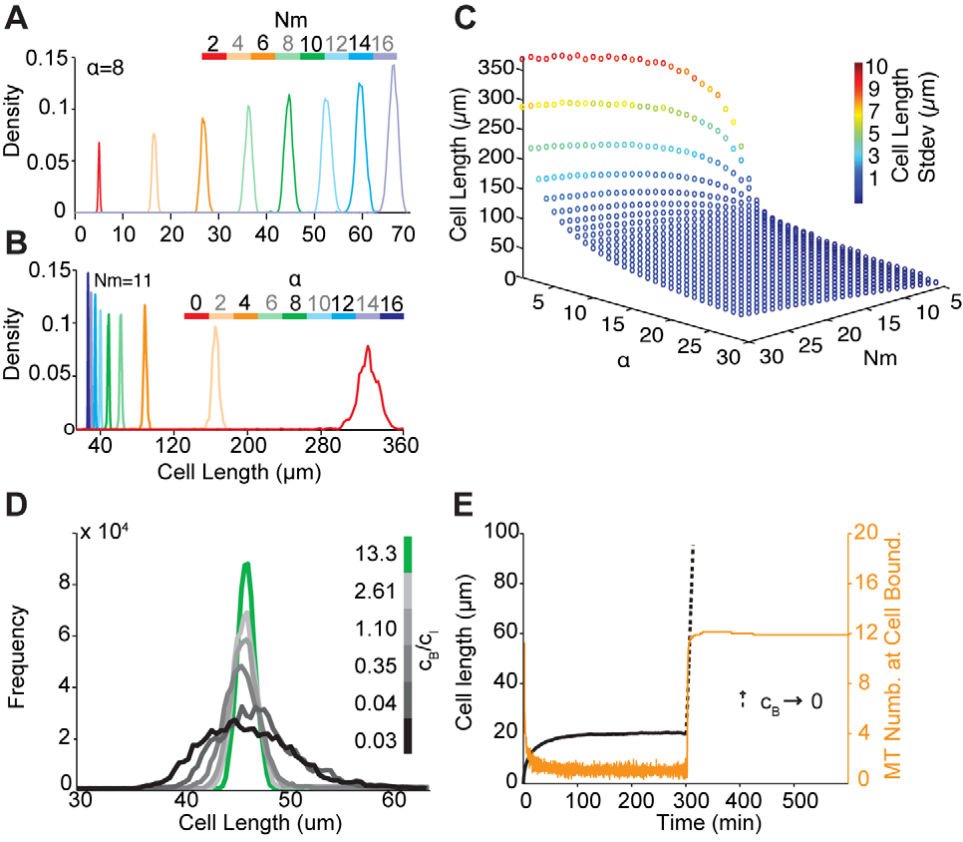
The role of parameters in the model on cell length homeostasis. (A) Cell length distributions were calculated from simulations using parameter values shown in Table 1 and α = 8 for different numbers of microtubules (*N*_m_ = 2–16). (B) Cell length distributions were calculated from simulations using parameter values shown in Table 1 and *N*_m_ = 11 for different levels of microtubule cooperation (α = 0–16). (C) To more fully explore the effects of different values of *N*_m_ and α (other model parameters as in Table 1) on cell length homeostasis, variation in cell length (standard deviation values are shown in colour using the colour-map shown) was measured over 10,000 min of simulation time. Homeostasis is achieved for a broad range of parameters above a low threshold value of α. (D) Cell length distributions with same mean values were calculated in the model for different values of *c*_B_/*c*_I_ from model simulations with α = 8 and *N*_m_ = 11, using *v*_g_ and *v*_B_ values shown in Table 1. (E) In a cell at steady state, a dramatic reduction in the value of the cell boundary catastrophe rate, *c*_B_, produces a dramatic increase in the steady-state number of microtubules (MT) at cell boundary (in orange) and a steady increase in cell length over time (black dotted line). α = 8 and *N*_m_ = 12; model parameters *c*_I_, *v*_g_, and *v*_B_ as shown in Table 1.

To test whether the dynamic behaviour of this oriented array of microtubules could define cell length in this system, we generated a simple mathematical model of microtubule-based cell length control (Figures 7 and 8). In contrast with previous models of cell spreading [10],[41],[42], our model was constructed to assess the effects of a polarised array of dynamic microtubules on cell elongation and homeostasis. Based upon our analysis of the cellular distribution of microtubules (Figures 5 and 6), EB3-GFP comets (Figure 6D), and γ-tubulin (Figure S2), microtubules in the model were assumed to grow out from the cell centre towards cell tips (Figure 7A). Cycles of dynamic instability were then implemented using values of growth and catastrophe rates taken from the experimental literature [43]. The interaction between microtubules and the cell cortex was then modelled by assuming that contact (i) increases the microtubule catastrophe rate (by a factor of 16 [43]) and (ii) drives the extension of the cell margin [44]. (Although the mechanism by which this occurs is not specified in the model, we think it likely that it is through the delivery of new material required for local growth [24] rather than through force generation [45],[46].) A slow fixed rate of margin retraction was then implemented, based upon measurements of the rate of retraction of cells on lines in the absence of microtubules (0.4±0.3 µm/min), which may reflect the turnover of material from the cell periphery. Simulations using this simple scheme were found to recapitulate the path of cell elongation and cell length homeostasis (Figure 7A–7C). Furthermore, the model predicts a linear decrease in microtubule density from the cell centre to the cell edge, which was verified experimentally (Figure 7D). Interestingly, the model also revealed that, irrespective of the actual cell length, it is the small number of dynamic microtubules (approximately two) that reach the cell cortex that maintain cell length homeostasis, by countering the tendency of the cell margin to retract (Figures 7C and S4). This is in line with previous data suggesting that a small population of pioneer microtubules is sufficient in some systems to drive forward movement of the cell edge [47].

**Figure 9:**
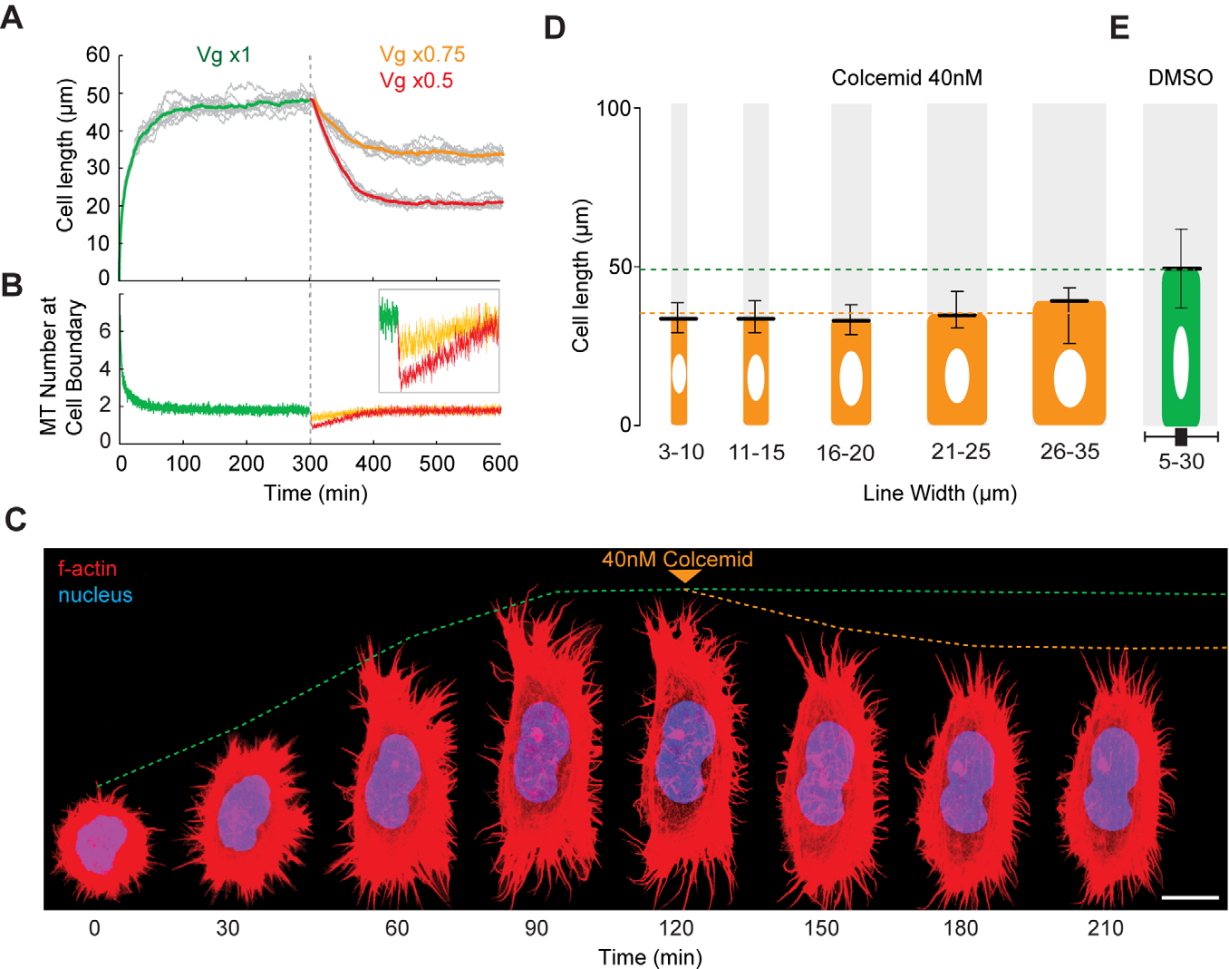
Testing the model of microtubule-based cell length homeostasis. (A and B) Model cell length dynamics (A) and numbers of microtubules (MT) interacting with the cell margin (B) were analysed as the microtubule polymerisation rate, *v*_g_, was changed by a factor of 0.75 (orange) or 0.5 (red). (*N*_m_ = 11 and α = 8; other parameters were fixed.) (C–E) As a test of this model, 40 nM colcemid (D) or an equivalent volume of DMSO (E) was added to HeLa cells on lines of varying widths (3–10 µm, 11–15 µm, 16–20 µm, 21–25 µm, and 26–35 µm) at steady state, and average cell length was measured. An HeLa cell spreading on a fibronectin line is shown (C) before and after treatment with 40 nM colcemid (at 120 min, indicated by orange arrow). The green dotted line indicates expected length without perturbation. Orange dotted line indicates measured cell length. Scale bar = 10 µm. Error bars denote upper and lower box plot quartiles.

When we examined the effects of varying the remaining free parameters in the model (Figure 8A–8C), we found that cell length homeostasis (the coefficient of variation in cell length) was marginally sensitive to changes in the value of α, representing the level of microtubule cooperation in the system (Figure 8A–8C), and to changes in *N*_m_, the number of cooperating microtubules. Based on measurements of cell length and microtubule numbers in HeLa cells on lines, we were able to estimate the value of α as 8, implying that individual microtubules cooperate to drive cell spreading. More significantly, an analysis of the effects of varying the other experimentally determined parameters in the model revealed that cell length control critically requires a high value of *c*_B_/*c*_I_, the ratio of the microtubule catastrophe rate at the cortex (*c*_B_) to that in the cell interior (*c*_I_). Thus, cell length becomes progressively more variable as the ratio of *c*_B_/*c*_I_ is reduced, e.g., as *c*_B_ tends to 0 (Figures 8D, 8E, and S4B). By contrast, changes in the microtubule polymerisation rate (*v*_g_) induce corresponding changes in cell length in the model without inducing a loss of homeostasis (Figures S4A, 9A, and 9B), causing the system to stabilise at a new steady-state length when the number of microtubule plus ends interacting with the cell cortex returned to the equilibrium value of ∼2 (Figure 9B). This serves as a good test of the likely effects of the addition of a “microtubule inhibitor” on cell length control (Figure 9A and 9B). To test whether this prediction is borne out in experiment, we used an inhibitor of microtubule dynamics [38] to reduce the rate of microtubule polymerisation in HeLa cells on lines of varying width at steady state. After allowing cells 2 h to spread, 40 nM colcemid or an equivalent amount of the carrier DMSO was added to the medium for 30 min (Figure 9C–9E). In the case of colcemid, but not DMSO, this was sufficient to disturb microtubule organisation without causing a complete loss of microtubules (data not shown), and induced active cell shortening (Figure 9C and 9D). As predicted, cells settled down to a new shorter length following this treatment, irrespective of line width (Figure 9C and 9D). We conclude that the dynamic behaviour of the population of longitudinally polarised microtubules plays a key role in homeostatic cell length control.

### An Oriented Population of Microtubules Controls Epithelial Height in the Developing Zebrafish Neural Tube

**Figure 10:**
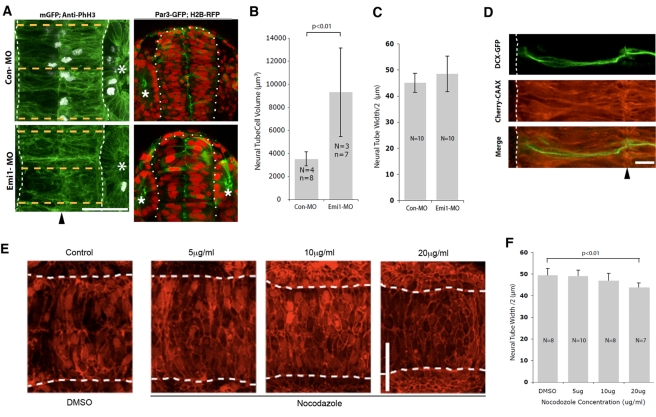
Zebrafish neural tube width is not affected by increases in cell volume but is sensitive to low levels of nocodazole. (A) In the left panels neural tubes (outlined with white dots) were visualised with membrane GFP (mGFP), imaged from dorsal view, and measured at three positions (orange dotted lines) around the otic vesicle (asterisk), and the measurements were averaged. Lack of anti-phospho-histone H3 (anti-PhH3) staining in lower panel indicates that Emi1-MO effectively blocks cell divisions. (Midline of neural tube indicated by black arrowhead. Scale bar = 50 µm.) In right panels embryos are expressing Par3-GFP to reveal the nascent apical surface of the neural tube and H2B-RFP to reveal the enlarged nuclei of the division blocked in *emi1* morphant embryos (sections viewed in near transverse plane). (B) Cell volume is significantly larger in *emi1* morphants (Emi1-MO) than it is in control embryos (Con-MO). (C) Neural tube width was not significantly different between Con-MO and Emi1-MO embryos. (D) Zebrafish embryos were injected with a plasmid expressing DCX-GFP to visualise the microtubule cytoskeleton and mRNA encoding Cherry-CAAX to visualise the neural tube cells. Microtubule bundles were present along the entire length of labelled cells. (Midline indicated by black arrowhead. Scale bar = 10 µm.) (E) Neural tube structure is not affected by treatment with low levels of nocodazole. Zebrafish embryos were injected with mRNA encoding Cherry-CAAX and H2B-RFP and treated with nocodazole at different concentrations. Scale bar: 50 µm. (F) The neural tube of nocodazole-treated embryos was narrower than that of control embryos, and epithelial width was reduced in a concentration-dependent manner.

Previous work has suggested roles for microtubules in the regulation of cell shape in 3-D environments [48]. Therefore, we were prompted to test whether cells exhibit a similar type of cell length homeostasis in a tissue and developmental context. We used the zebrafish neural tube as a simple model system for this analysis for several reasons. First, cells in this tissue are bipolar in form and of similar length to S2R+ and HeLa cells on lines. Second, once formed [16], this tissue is maintained as a stable structure consisting of two parallel columns of highly elongated neuroepithelial cells [49], making reliable measurements of cell length relatively easy. Third, the tissue is amenable to imaging and perturbation experiments using morpholinos. To begin, we tested whether cell length depends upon cell volume in the zebrafish neural tube by arresting cells in the G2 phase of the cell cycle using an established protocol in which a morpholino against the translational start site of the G2/M regulator Emi1 (Emi1-MO) or a control morpholino (Con-MO) is injected into the one-cell embryo [50],[51]. As previously reported, this treatment does not affect cell division until the neural plate stage because of a maternal effect and has very limited cytotoxicity [50],[51]. For consistency across animals, measurements of cell lengths were then made using the neuroepithelium of the hindbrain close to the developing otic vesicle in 19 somite (19s)–stage embryos. At this stage in development the neural tube has not yet inflated its ventricle, and neuroepithelial cells from the left and right sides meet in the middle of the tube, as confirmed by the expression of the polarity protein Par3-GFP, which reveals the apical ends of all cells lining up along the tube midline in both control and *emi1* morphant embryos (Figure 10A). This block in cell cycle progression (evident in the loss of pH3 staining in Figure 10A) led to a significant 2.6-fold increase in neuroepithelial cell volume (Figure 10B; Emi1-MO, 9,034.2±3,839.3 µm^3^, *n* = 7 cells from three embryos; Con-MO, 3,516.4±608.2 µm^3^, *n* = 8 cells from four embryos; *t* test, *p* = 0.007) when compared to control-morpholino-injected embryos. The variability in the extent of volume increase observed likely reflects the slight variability of the timing with which the Emi1-MO-induced cell cycle arrest kicks in in different embryos.

Since the vast majority of individual cells in the tissue span the entire width of the neural tube (Figure 10A), we were able to use the width of the tissue at three locations close to the otic vesicle to estimate average cell length. Despite the large difference in cell volumes between control and *emi1* morphant embryos (readily visible in embryos labelled with Par3-GFP and H2B-RFP; Figure 10A), the length of neuroepithelial cells remained unaltered (Figure 10C; Con-MO, 44.97±3.66 µm, *n* = 10; Emi1-MO, 48.11±6.83, *n* = 10; *t* test, *p* = 0.188). This shows that cell length is independent of cell volume in this tissue context as it is in our cell culture models.

To test whether microtubule dynamics are required to define cell length in this system, we visualised microtubule cytoskeletal organisation within the neuroepithelium. Stochastic labelling of cells in the neural tube with GFP fused to the microtubule-associated protein doublecortin (DCX) [52] revealed bundles of parallel microtubules running the entire length of each neuroepithelial cell (Figure 10D), from the apical to the basal limit of the epithelium. To determine whether these microtubules function in cell length control, as was shown for microtubules in cells on micropatterned lines, we added low doses of the microtubule inhibitor nocodazole to these embryos for a period of 30 min. While high doses of microtubule inhibitors lead to a reversible loss of neuroepithelial form as all the cells in the tissue round up (data not shown), this treatment leaves overall tissue architecture intact (Figure 10E). The result was a significant reduction in the width of nocodazole-treated neural tubes when compared to those in DMSO control embryos (Figure 10F), without affecting differences in width that characterise different parts of the tissue. Moreover, as the concentration of nocodazole was increased, the neural tube became progressively narrower (DMSO, 49.37±3.11 µm, *n* = 8; 5 µg/ml nocodazole, 48.99±2.73 µm, *n* = 9; 10 µg/ml nocodazole, 46.93±3.29 µm, *n* = 8; 20 µg/ml nocodazole, 43.77±1.99 µm, *n* = 7). This analysis suggests that the parallel bundles of microtubules seen spanning the entire width of the epithelium within individual neuroepithelial cells play a critical role in the ability of cells to maintain their length and proper tissue architecture, as they do in homeostatic length control in cells in culture.

## Discussion

Taken together our analysis of hemocyte-derived *Drosophila* cells and epithelial-derived HeLa cells on micro-contact-printed lines in culture, and of neuroepithelial cells in the developing zebrafish neural tube, identifies a capacity for animal cells to maintain an intrinsically defined length. Cell length control in these systems appears to act independently of cell width and volume. Instead, it relies on the continuing presence of a polarised population of dynamic microtubules that, as a result of interactions with the cell cortex, come to lie parallel to the long axis of cells, running from the centre to either cell tip. Because cell extension depends on microtubules, this oriented array of dynamic microtubules is then in a position to regulate cell length. Somewhat surprisingly, we observed no role for actin-based cortical tension in opposing microtubule-driven cell elongation in our system. This is in line with data from previous studies showing that the final cell spread area is independent of the actin cytoskeleton [10],[24]. Because of this, cell length control is unlikely to reflect a balance of forces between contractile actin filaments and extending microtubule rods—as previously hypothesized for the regulation of animal cell form [22]. In fact, a recent study showed that microtubules do not bear a significant mechanical load in fully spread cells [53]. Based on these observations, it seems likely that microtubules drive cell elongation through the promotion of directional traffic of material from the Golgi to the cell surface [24], rather than through the direct generation of mechanical force itself. Interestingly, microtubule dynamics have also been shown to contribute to (i) the regulation of cell length and cell shape in fission yeast via the regulation of cell transport [54], (ii) axial elongation in plant cells [55], and (iii) the maintenance of spindle length within defined limits [56]. As such, limits to the length of dynamic microtubule-based structures may be a relatively widespread phenomenon in biology.

Recently, a mechanism for length-dependent microtubule depolymerisation was discovered [5], which could help to explain the regulation of processes such as cell length. While this mechanism may be involved in cell length control, our analysis suggests that it may not be necessary to invoke this type of control to understand the maintenance of microtubule-dependent structures within relatively well-defined size limits in all cases, since cell length control can arise as a relatively simple by-product of microtubule-based cell extension if a few simple propositions hold. These propositions are the following: (i) that dynamic microtubules are polarised so that they polymerise along the long cell axis towards cell tips, (ii) that the rate of microtubule catastrophe increases when microtubules reach the cell's ends, and (iii) that microtubules act together to prevent retraction of the cell margin and promote cell elongation, probably through the delivery of material to counter turnover of material at the cell tips. Significantly, a model based on these experimentally well-established assumptions predicts the spreading dynamics we observe on patterned lines, the steady-state microtubule distribution (Figure 7D), the path of cell elongation (Figures 9A, 9B, and S4B), and the effects of a microtubule poison on cell length (Figure 9C and 9D).

Intrinsic regulation of cell length in a tissue and developmental context is inherently difficult to demonstrate unambiguously because of the potentially confounding effects of forces and signals from neighbouring tissues and extracellular matrix. For example, tissue architecture and the dimensions of neuroepithelial cells in the newly formed zebrafish neural tube are likely to be influenced by both extrinsic and cell-intrinsic factors, as cells undergo interdigitation and intercalation across the midline [16],[57]. Moreover, zebrafish neuroepithelial cells achieve their final length through a complex mechanism involving initial overextension and then retraction (data not shown) as they establish a nascent apical domain at the tissue midline [16]. Because tissue architecture in this case is generated via a complex process of self organisation, an intrinsic mechanism that biases cell length may be indispensable to ensure robust organ size and form in the face of variations in the volume of component cells and variations in the movements and differentiation of surrounding tissues [58]. Indeed, here we show that neuroepithelial cell length in vivo is independent of cell volume but, consistent with our findings in vitro, dependent on a population of axially oriented microtubules. As a result, increasing levels of microtubule inhibitors administered over a relatively short period of time cause systematic reductions in epithelial height (Figure 10C). These data help to explain how it is that neuroepithelial height can remain relatively unchanged in ectopic neural tubes that are not situated at the embryonic midline in morphogenetic mutants (compare cells in Figure 4C and 4F in [16]; compare cells in Figure 1G, 1H, and, 1K in [57]).

Similar conclusions can be drawn from earlier studies on the effects of cell cycle arrest on development [59],[60], where it was noted that many ultrastructural features and functional properties of cells were conserved despite dramatic changes induced in cell size. Our study shows that the regulation of neuroepithelial height is one way by which embryos are able to do this. More generally, one could hypothesize that cell length homeostasis is likely to be required in all growing epithelia that need to maintain apical–basal structure despite continual changes in the volumes of their constituent cells.

While the emphasis of this study is cell length homeostasis, it is clear that changes in animal cell length or height are likely to play a critical role during tissue morphogenesis in vivo. During *Drosophila* gastrulation, for example, epithelial cells are thought to actively contribute to ventral furrow formation by undergoing apical constriction and cell shortening [15], a process that would seem to require orchestrated changes in cell length. Although the mechanism by which this occurs is not understood, it is plausible that microtubules, which are highly polarised along the apical–basal cell axis of *Drosophila* epithelial cells, play a role, as has been shown in other epithelia [61],[62]. Similarly, the changes in cell length that accompany neuron and myoblast differentiation are brought about by changes in microtubule dynamics [63]–[65]. Thus, many animal cells are likely to need to be able to regulate their optimal steady-state length, e.g., by changing the rate of microtubule polymerisation to alter their resting length whilst preserving length control (Figures 9A and S4A). Conversely, decreasing the susceptibility of microtubules to undergo catastrophe at the cell cortex, e.g., by crosslinking microtubules with Map1A [63], could induce dramatic but relatively unregulated cell elongation (Figure 8E). Because of this, long cells like neurons may rely on additional control systems, like length-dependent regulators of microtubule catastrophe [5] or environmental cues, to reach specific locations during the process of axon pathfinding. An important goal of future research will be to identify how intrinsic length constraints and additional layers of control are altered during animal development to give specific cells and tissues their characteristic forms, and to determine whether the deregulation of cell length control contributes to the loss of tissue homeostasis seen in diseases such as cancer.

## Materials and Methods

### Cell Culture Methods

#### Micro-contact printing and cell patterning

Micro-contact printing was carried out as described previously [66]. A master (or template) was used to generate a polydimethylsiloxane (PDMS) stamp. Briefly, the master was fabricated as follows. In a clean room, a 4-in. silicon wafer was cleaned under nitrogen and spin-coated at 3,000 rpm for 20–30 s with 2 ml of S1818 (Shipley) positive resist to obtain a wafer with a ∼2-µm-thick resist coating. The wafer was then baked on a hot plate at 100°C for 1 min and the positive resist exposed to 6 mW/cm^2^ UV light at 405 nm (KARL SUSS aligner MJB 3UV 300) for 30–50 s. The exposed areas of the S1818 positive resist were then removed by dipping the wafer in a solution of microposit developer (35i, Shipley). The wafer was manually agitated for ∼1 min and rinsed using de-ionised H_2_O. The developing procedure was repeated three times and the master dried under a stream of nitrogen gas, before being baked again for 1 min on a hot plate at 100°C. The PDMS stamps (Sylgard kit, Dow Corning) were made by using a previously described method [66]. The PDMS stamps were cleaned by sonication in 70% EtOH for 5 min, rinsed with de-ionised H_2_O, under a stream of nitrogen gas, and inked for 10 min in a solution of 10 µg/ml fibronectin (Sigma-Aldrich) together with 10 µg/ml fibronectin-FITC (Sigma-Aldrich) conjugated in PBS, or in a solution of 5 µg/ml ConA together with 5 µg/ml ConA-FITC (Sigma-Aldrich) conjugated in distilled water (for S2R+ cell patterning). After inking, PDMS stamps were dried under nitrogen gas and placed in contact with a glass coverslip (VWR International) that had been plasma-cleaned and coated with non-adhesive polyethylene glycol, PLL-g-PEG (SUSOS, Switzerland), for 1 min.

Before patterning, cells were washed in fresh serum-free medium (M3 at 24°C for *Drosophila* S2R+ cells and DIMEM at 37°C for HeLa cells). Cells were trypsinized (using Trypsin-EDTA, Invitrogen) and their volume assessed in a Coulter Counter (Multisizer II, Beckman-Coulter) at a 1∶10 dilution in Isoton II (Beckman-Coulter). The remainder were resuspended in serum-free medium at a density of 10^4^ cells/cm^2^ and seeded on the micropatterned coverslips. Typically, cells attach to the printed ConA or fibronectin within 10 min of seeding. After 30 min, non-adherent cells and debris were removed by replacing the medium with fresh serum-free medium. Serum-free medium was used to avoid the effects of ECM proteins in serum.

#### Cell culture

*Drosophila* S2R+ cells [67] were cultured in M3 media (Sigma-Aldrich, S3652) with 10% heat-inactivated fetal bovine serum (12103-78P, JRH Biosciences) as monolayers at room temperature (22–25°C) in treated culture flasks (Falcon, BD Biosciences). HeLa cells were cultured in Dulbecco's Modified Eagle Medium (DMEM) supplemented with 10% heat-inactivated fetal bovine serum and 1% Pen-Strep (Sigma-Aldrich). *Drosophila* cells were propagated and treated with dsRNA as previously described [37],[68]. For SCAR/WAVE dsRNA synthesis, primer sequences flanked with T7 sites were chosen for amplification of 300–600 bp of exonic sequence with smaller than 21-bp stretches of identity with any other gene. PCR products were then used as a template for the MEGAscript T7 reaction. RNAi was performed on S2R+ cells cultured in four-well tissue culture plate. Five days after RNAi, cells were gently removed from the plate, resuspended in Sang M3 Insect media (S3652, Sigma-Aldrich) and seeded on ConA patterns as described above. Small HeLa cells were obtained by using cells close to 100% confluency (measured by Coulter Counter, Multisizer II, Beckman-Coulter). For all other experiments, we used HeLa cells growing at 50% confluency (2 d after splitting a confluent culture 1∶10).

#### Drug treatment

In order to destabilise microtubules in cells in culture, 40 nM or 1 µM demecolcine (colcemid, Sigma-Aldrich, in DMSO) was added to HeLa cells on lines 90 min after seeding. A 40-nM dose was sufficient to alter microtubule organisation without altering microtubules organisation. A 1-µM dose was sufficient to depolymerise all microtubules. An equivalent amount of DMSO was used in control experiments. To alter cortical contactile forces, 40 µM blebbistatin, a non-muscle Myosin II inhibitor (B0560, Sigma-Aldrich) [33], was added to 50% confluent culture of HeLa cells. After 30 min of drug incubation the cells were gently trypsinized, and resuspended in DMEM with 40 µM blebbistatin, after 20 min cells were plated onto the micropatterned lines. An equivalent amount of DMSO was used in control experiments. After prolonged periods of time, this led to a majority of multinucleate cells as expected as the result of defects in actin-myosin-based cytokinesis (data not shown). The destabilisation of the actin lamellipodium in HeLa cells was achieved using NSC23766 (Calbiochem) [32], a Rac inhibitor. NSC23766 (40 µM) was added to 50% confluent culture of HeLa cells. After 30 min of drug incubation the cells were gently trypsinized and resuspended in DMEM with 40 µM NSC23766 and after 20 min plated onto the micropatterned lines. An equivalent amount of methanol was used in control experiments.

#### Immunofluorenscence microscopy

HeLa cells stably expressing live-act-GFP [69] were used for live-imaging experiments. For imaging actin filament organisation, S2R+ and HeLa cells were rinsed in PBS, fixed with 4% paraformaldehyde at room temperature, washed with PBS, permeabilized with 0.1% Triton X-100 in PBS, blocked for 1 h with 5% BSA in PBS, and stained with phalloidin-TRITC (1/400, Sigma-Aldrich), 4′-6-diamidino-2-phenylindole (DAPI) (1/1,000, Sigma-Aldrich), and a FITC-conjugated GM1α anti-α-tubulin antibody (1/400, Sigma-Aldrich). After staining, coverslips were washed in PBS, mounted on microscope slide under 20 µl of reagent (FluorSave, Calbiochem), and dried at 40°C for 1 h. To better visualise the microtubule organisation, cells were fixed with −20°C 100% methanol for 4 min. After 4 min, cells were rinsed in PBS, and microtubules immune-labelled using the FITC-conjugated GM1α anti-α-tubulin antibody. To visualise microtubule plus ends, HeLa and S2 cells were transfected with EB3-GFP and EB1-GFP, respectively, and, analysed 48 h after transfection. Chemically fixed and live cells were imaged using a Leica SP5 inverted confocal microscope or a Nikon TE2000 inverted microscope.

#### Image and data analysis

Images of cells on lines were acquired automatically using a 20×/0.45 N.A. objective on a Nikon TE2000, and Metamorph software (Universal Imaging) to drive an automated Prior stage. Cell length and microtubule polarity data were measured using in-house automated software that was designed to remove user bias and to analyse large datasets. For the measurement of cell length (Figure S1C), images of cells were thresholded for segmentation, mononucleate cells selected, and maximum cell length parallel to the line measured. Pattern width (Figure S1B) was calculated empirically from images of fluorescent lines in each case. To analyse microtubule organisation, grayscale images of cells were processed using a median filter and thresholding. The probably of finding microtubule polymer oriented in a given direction in Figure 5 was determined by normalising the amount of linear tubulin polymer at that angle by the total amount of linear polymer in the cell. In-house software was developed in Java (http://www.java.com) and integrated in ImageJ (http://rsbweb.nih.gov/ij/). The data analysis was mainly performed using R environment (http://www.r-project.org/) and Matlab.

### Mathematical Model of Microtubule-Based Cell Extension

A mathematical model was formulated to examine the likely role of microtubules in the control of cell length. The model was based on experimental observations that microtubules aligned along the long axis of the cell, with plus ends towards the cell tips (Videos S1, S2, S3), appear to drive the elongation of cell edges on adhesive lines (Figure 4A–4F) and in cells lacking lamellipodia (Figure 3). Using this as a framework, we constructed a simple stochastic half-cell model of cell elongation driven by a population of parallel dynamic microtubules. Where possible, parameters used to model microtubule dynamics were taken directly from experimental data (see Table 1). We then made a number of simplifying assumptions. First, we assumed that a fixed number of microtubules, *N*_m_, nucleate at the cell centre and grow towards the cell ends at a rate determined by the known rate of microtubule polymerisation, *v*_g_, with an experimentally defined cytoplasmic catastrophe rate *c*_I_ [43], and we made the simplifying assumption that there is no rescue of microtubule growth following catastrophe. Upon reaching the cortex (defined as the region within 3 µm of the cell boundary), microtubule plus ends then act together to promote extension of the cell boundary. We modelled the cooperative effects of *n* microtubules touching the cortex in driving cell elongation at each time step using the function *e* ^−α/*n*^. Because microtubules can drive cell elongation through forces generated by the addition of tubulin subunits [70], through the delivery of new material required for local growth, and/or through local modification of the cell cortex [45],[46], in this study α is assumed to be a free, dimensionless parameter. At the same time, in line with cell biological data, contact with the cortex induces an increase in the rate of microtubule catastrophe *c*_B_ [43] (See Table 1). Since catastrophe events free up a microtubule nucleation site in the model, the number of growing microtubules remains constant over time. Finally, the term *v*_B_ was added, based upon experimental data (data not shown), to represent the slow retraction of the cell margin in the absence of microtubules (0.4±0.3 µm/min). After applying known rates of microtubule growth and catastrophe to the model (see Table 1), two free variables remain: α, which governs the cooperative effect of microtubules on the movement of the cell boundary, and *N*_m_, the number of microtubule nucleation sites. In order to compare the results of simulations with experimental data from Figures 1–3, simulations were run in Matlab by using parameter values shown in Table 1, and the position of the boundary was used as a read out of half cell length.

**Table 1:** Model parameters.

Parameter Type	Parameter	Symbol	Value	Unit
**Fixed**	Microtubule growth velocity^a^	*v*_g_	15±4	µm min^−1^
	Microtubule catastrophe internal rate^a^	*c*_I_	0.3	min^−1^
	Microtubule catastrophe rate at cell boundary^a^	*c*_B_	4.8	min^−1^
	Cell boundary retraction rate^b^	*v*_B_	0.4±0.3	µm min^−1^
**Free**	Total number of nucleation sites	*N*_m_	>0	
	Cooperative behaviour of individual microtubules to cell elongation constant	α	>0	

a Values from [43].

b Values from experimental data in this study.

Based on this scheme, given a random number *r* in the interval [0,1], the stochastic equations that describe the microtubule growth in time are given by
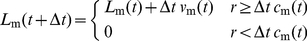
(1)

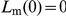
Where *L*_m_ is the microtubule length, *r* is a random number in the interval 0≤*r*≤1, Δ*t* (0.001 min) is the simulation time step, and *v*_m_ and *c*_m_ are the microtubule velocity and catastrophe rates, respectively. The stochastic catastrophe event is defined by the random number *r* and Δ*t c*_m_. In order to model cell extension, a cell length boundary equation *L*_B_ is added to the model:
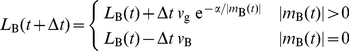
(2)

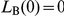
where *v*_g_ is the microtubule growing rate internal to the cell and *v*_B_ is the cell boundary velocity retraction rate when there are no microtubules crossing the boundary. α governs the cooperative effect of microtubules in promoting cell boundary extension, as defined by the function e^−α/|*m*_B_^ ^(*t*)|^. *m*_I_ and *m*_B_ describe internal and boundary microtubules as follows:
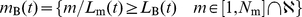
(3)

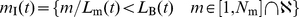
(4)Finally the general microtubule velocity and catastrophe rate equations are given by
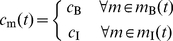
(5)

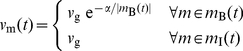
(6)where *c*_I_ and *c*_B_ are the internal and boundary catastrophe microtubules rates, respectively.

The aim of our model was to quantitatively explore the relationship between microtubule behaviour and cell length control. Significantly, for a wide range of *N*_m_ and α values, this simple scheme recapitulated the path of cell elongation and length control seen in observations of cells on lines (Figure 1). As seen in experiments (Figure 1D), the rate of cell elongation in the model diminished over the course of 60 min, leading to a steady-state cell length within a few hours (Figure 7B). To understand the source of cell length homeostasis in these simulations we plotted the number of microtubules contacting the cell cortex over time (Figure 7C). This revealed a steady decrease in the number of microtubules reaching the cell cortex as cells elongate. As cells extend, this number plateaus, reaching a steady equilibrium between cell elongation and cell retraction that maintains cell length over time, which is typically approximately two microtubules—a number that is independent of the number of microtubule nucleation sites and cell length itself (Figure 7C). The model also predicted a linear decrease in microtubule density with distance from the cell centre similar to that measured in cells on lines (Figure 7D).

Significantly, the number of microtubule nucleation sites, *N*_m_, had little impact on the ability of cells to achieve length homeostasis (Figure 8A and 8B; cell length variance is used as quantitative measure of homeostasis), while a moderate level of microtubule cooperation (α>3) was required for a reproducible cell length (Figure 8B and 8C). Above this threshold, while cells were able to maintain a homeostatic cell length irrespective of the specific values of *N*_m_ and α, cell length increased with increasing values of *N*_m_ and decreased with increasing values of α (Figure 8C).

Although catastrophe rates used in the model were based on experimentally well-defined parameters, we also determined the effects of changing the cortical *c*_B_ and internal *c*_I_ catastrophe rates on cell length homeostasis. This revealed that cell length control is gradually lost when *c*_B_/*c*_I_ tends to zero, i.e., as *c*_B_ values were reduced or *c*_I_ increased. This is seen by the increase in cell length variance—a quantitative measure of homeostasis (Figures 8D and S4B). It should also be noted that at values of *c*_B_ close to zero, the number of microtubules at the cell boundary increases to a high steady-state value, driving continuous cell elongation (Figure 8E). Finally, the model was used to test the likely effects of colcemid in this system [38] by altering *v*_g_. A reduction in *v*_g_ leads to a linear reduction in cell length (Figure S4A), as cells re-establish equilibrium with an average of approximately two microtubules contacting the cell cortex per unit time (Figure 9A and 9B).

### Zebrafish Methods

#### Cell cycle block and neural tube width measurements

To block cell division, zebrafish embryos were injected with 1 nl of 0.5 mM antisense morpholino against the start site of the early mitotic inhibitor 1 (Emi1-MO) [50],[51]. Standard control morpholino (Con-MO) (Genetools) was injected at the same concentration. To monitor the efficiency of the division block, embryos were immunostained with antibody against phospho-histone H3 (Upstate Biologicals).

To visualise neural tube organisation, embryos were injected with either mRNA (100 pg) encoding membrane-GFP, or Par3-GFP and H2B-RFP. Capped mRNA was synthesised with mMessage Machine kit (Ambion).

Neural tube cell volumes were calculated assuming the cell is a block, with height being the length of the long side of the cell, width being the length of the short side of the cell, and depth calculated from the number of confocal sections occupied by the cell. Measurements were taken from images of mosaically labelled 19s embryos injected with mRNA encoding a membrane marker (mGFP or Cherry-CAAX) and H2B-RFP at 32- to 64-cell stage. To measure the width of the neural tube, embryos were fixed at 19s, and confocal images of the hindbrain were taken from dorsal to ventral. To adjust for tilting of the neural tube, 3-D projection of the neural tube was generated using the 3D Projection Tool in ImageJ. Width of the neural tube was measured at three positions around the otic vesicle and averaged (Figure 10A).

#### Microtubule visualisation and perturbation

To visualise microtubules, zebrafish embryos were injected with plasmid expressing DCX-GFP (generous gift from Steve Wilson, University College London). DCX-GFP was injected with mRNA encoding Cherry-CAAX and H2B-RFP to visualise cell outlines (data not shown). For drug treatment, nocodazole was dissolved in fish water from concentrated stock (5 mg/ml DMSO), and DMSO at 0.4% was used as vehicle control. The 19s embryos were then soaked in this medium for 30 min at 28°C before being fixed. Neural tube width was measured as previously described.

## Supporting Information

Figure S1 **Image analysis of patterned cells.** (A) Images of TRITC-labelled F-actin in cells on lines were acquired in a vertical orientation. (B) The pattern width was calculated by averaging the widths obtained at the intersection of a scanning horizontal line (blue line) with a binary pattern image (in black) as the scanning line moves vertically. (C) Lengths of patterned cells were obtained by fitting a rectangular shape to binary images of cells stained for F-actin and taking the length of the rectangle side parallel to the patterned line (yellow line). Lengths of non-patterned cells were obtained by taking the greatest distance possible between any two points along the cell boundary (Feret's diameter) (see Figure 1B). Only the length of mononuclear cells was measured. The cell ([A], yellow dotted) and the line pattern boundaries ([A], blue dotted) were obtained by thresholding image intensity. The cell and pattern boundary were successively filled inside and outside by black and white, respectively, to obtain the binary images shown in (B) and (C). Cell nucleus was segmented by the same method.
(0.51 MB TIF)

Figure S2 **Microtubule nucleation.** Microtubule nucleation sites in HeLa cells were labelled by γ-tubulin first antibody (A) and Alexa Cy5 secondary antibody (B). Microtubules were labelled by FITC-conjugated anti-α-tubulin antibody (C). Nucleus was labelled by DAPI (D).
(2.53 MB TIF)

Figure S3 **Dynamic microtubules lie along the cell bottom, close to the substrate.** (A) Dynamic microtubules are concentrated within 1 µm of the substrate. Confocal *zy*-section projection of an HeLa cell transfected by EB3-GFP on a non-patterned fibronectin surface (B) and on a patterned fibronectin line (C). Top (D and E) and bottom (F and G) part of each HeLa cell transfected by EB3-GFP.
(0.26 MB DOC)

Figure S4 **The effects of microtubule polymerisation rates on average cell length.** (A) The microtubule growth rate (*v*_g_) in the model was plotted against steady-state values of cell length. The model predicts a linear relationship between microtubule polymerisation rate and cell length at steady state (*y* = 1.7*x*−2). (B) Variations in cell length (standard deviation) are shown for cells ∼44 µm long following changes in the catastrophe rates of microtubules at the cell boundary, *c*_B_ (in orange), or of cytoplasmic microtubules, *c*_I_ (in green). Average values were obtained by examining the variance when length was chosen to be close to experimentally determined values, using values of *N*_m_ and α varying from 1 to 30 and from 0 to 30, respectively, while other parameters were kept constant (with values specified in Table 1).
(2.58 MB TIF)

Video S1 **Microtubule plus ends in an HeLa cell on a thin micropatterned line.** EB3-GFP-labelled microtubule plus ends in an HeLa cell on a thin fibronectin patterned line (not shown).
(1.33 MB MOV)

Video S2 **Microtubule plus ends in an HeLa cell on a thick micropatterned line.** EB3-GFP-labelled microtubule plus ends in an HeLa cell on a thick fibronectin patterned line (not shown).
(0.97 MB MOV)

Video S3 **Microtubule plus ends in an S2 cell on a micropatterned line.** EB1-GFP-labelled microtubule plus ends in an S2 cell on a ConA patterned line (not shown).
(0.61 MB MOV)

## Abbreviations

19s: 19 somite
ConA: Concanavalin A
DAPI: 4′-6-diamidino-2-phenylindole
DCX: doublecortin
DMEM: Dulbecco's Modified Eagle Medium
dsRNA: double-stranded RNA
PDMS: polydimethylsiloxane
RNAi: RNA interference

## References

[1] Wemmer K. A, Marshall W. F (2007). Flagellar length control in chlamydomonas—paradigm for organelle size regulation.. Int Rev Cytol.

[2] Jorgensen P, Tyers M (2004). How cells coordinate growth and division.. Curr Biol.

[3] Castillo A, Nowak R, Littlefield K. P, Fowler V. M, Littlefield R. S (2009). A nebulin ruler does not dictate thin filament lengths.. Biophys J.

[4] Wühr M, Chen Y, Dumont S, Groen A. C, Needleman D. J (2008). Evidence for an upper limit to mitotic spindle length.. Curr Biol.

[5] Varga V, Leduc C, Bormuth V, Diez S, Howard J (2009). Kinesin-8 motors act cooperatively to mediate length-dependent microtubule depolymerization.. Cell.

[6] Foethke D, Makushok T, Brunner D, Nédélec F (2009). Force- and length-dependent catastrophe activities explain interphase microtubule organization in fission yeast.. Mol Syst Biol.

[7] Martin S. G (2009). Microtubule-dependent cell morphogenesis in the fission yeast.. Trends Cell Biol.

[8] Echave P, Conlon I. J, Lloyd A. C (2007). Cell size regulation in mammalian cells.. Cell Cycle.

[9] Tzur A, Kafri R, LeBleu V. S, Lahav G, Kirschner M. W (2009). Cell growth and size homeostasis in proliferating animal cells.. Science.

[10] Cuvelier D, Théry M, Chu Y, Dufour S, Thiéry J (2007). The universal dynamics of cell spreading.. Curr Biol.

[11] Keren K, Pincus Z, Allen G. M, Barnhart E. L, Marriott G (2008). Mechanism of shape determination in motile cells.. Nature.

[12] Lacayo C. I, Pincus Z, VanDuijn M. M, Wilson C. A, Fletcher D. A (2007). Emergence of large-scale cell morphology and movement from local actin filament growth dynamics.. PLoS Biol.

[13] Levina E. M, Kharitonova M. A, Rovensky Y. A, Vasiliev J. M (2001). Cytoskeletal control of fibroblast length: experiments with linear strips of substrate.. J Cell Sci.

[14] Kharitonova M. A, Vasiliev J. M (2008). Controlling cell length.. Semin Cell Dev Biol.

[15] Leptin M (1999). Gastrulation in Drosophila: the logic and the cellular mechanisms.. EMBO J.

[16] Tawk M, Araya C, Lyons D. A, Reugels A. M, Girdler G. C (2007). A mirror-symmetric cell division that orchestrates neuroepithelial morphogenesis.. Nature.

[17] Chen C. S, Mrksich M, Huang S, Whitesides G. M, Ingber D. E (1998). Micropatterned surfaces for control of cell shape, position, and function.. Biotechnol Prog.

[18] Dunn G. A, Heath J. P (1976). A new hypothesis of contact guidance in tissue cells.. Exp Cell Res.

[19] Dunn G. A, Brown A. F (1986). Alignment of fibroblasts on grooved surfaces described by a simple geometric transformation.. J Cell Sci.

[20] Oakley C, Brunette D. M (1993). The sequence of alignment of microtubules, focal contacts and actin filaments in fibroblasts spreading on smooth and grooved titanium substrata.. J Cell Sci.

[21] Yanagawa S, Lee J. S, Ishimoto A (1998). Identification and characterization of a novel line of Drosophila Schneider s2 cells that respond to wingless signaling.. J Biol Chem.

[22] Ingber D. E (2003). Tensegrity I. Cell structure and hierarchical systems biology.. J Cell Sci.

[23] Littlefield R. S, Fowler V. M (2008). Thin filament length regulation in striated muscle sarcomeres: pointed-end dynamics go beyond a nebulin ruler.. Semin Cell Dev Biol.

[24] Gauthier N. C, Rossier O. M, Mathur A, Hone J. C, Sheetz M. P (2009). Plasma membrane area increases with spread area by exocytosis of a GPI-anchored protein compartment.. Mol Biol Cell.

[25] Domnina L. V, Rovensky J. A, Vasiliev J. M, Gelfand I. M (1985). Effect of microtubule-destroying drugs on the spreading and shape of cultured epithelial cells.. J Cell Sci.

[26] Vasiliev J. M, Gelfand I. M, Domnina L. V, Ivanova O. Y, Komm S. G (1970). Effect of colcemid on the locomotory behaviour of fibroblasts.. J Embryol Exp Morphol.

[27] Bliokh Z. L, Domnina L. V, Ivanova O. Y, Pletjushkina O. Y, Svitkina T. M (1980). Spreading of fibroblasts in medium containing cytochalasin B: formation of lamellar cytoplasm as a combination of several functional different processes.. Proc Natl Acad Sci U S A.

[28] Giannone G, Dubin-Thaler B. J, Döbereiner H, Kieffer N, Bresnick A. R (2004). Periodic lamellipodial contractions correlate with rearward actin waves.. Cell.

[29] Baum B, Cherbas L (2008). Drosophila cell lines as model systems and as an experimental tool.. Methods Mol Biol.

[30] Hall A (2005). Rho GTPases and the control of cell behaviour.. Biochem Soc Trans.

[31] Kunda P, Craig G, Dominguez V, Baum B (2003). Abi, Sra1, and Kette control the stability and localization of SCAR/WAVE to regulate the formation of actin-based protrusions.. Curr Biol.

[32] Gao Y, Dickerson J. B, Guo F, Zheng J, Zheng Y (2004). Rational design and characterization of a Rac GTPase-specific small molecule inhibitor.. Proc Natl Acad Sci U S A.

[33] Straight A. F, Cheung A, Limouze J, Chen I, Westwood N. J (2003). Dissecting temporal and spatial control of cytokinesis with a myosin II inhibitor.. Science.

[34] Döbereiner H, Dubin-Thaler B. J, Giannone G, Sheetz M. P (2005). Force sensing and generation in cell phases: analyses of complex functions.. J Appl Physiol.

[35] Kharitonova M. A, Vasiliev J. M (2004). Length control is determined by the pattern of cytoskeleton.. J Cell Sci.

[36] Winckler B, Solomon F (1991). A role for microtubule bundles in the morphogenesis of chicken erythrocytes.. Proc Natl Acad Sci U S A.

[37] Kiger A. A, Baum B, Jones S, Jones M. R, Coulson A (2003). A functional genomic analysis of cell morphology using RNA interference.. J Biol.

[38] Vandecandelaere A, Martin S. R, Engelborghs Y (1997). Response of microtubules to the addition of colchicine and tubulin-colchicine: evaluation of models for the interaction of drugs with microtubules.. Biochem J.

[39] Waterman-Storer C. M, Salmon E. D (1997). Actomyosin-based retrograde flow of microtubules in the lamella of migrating epithelial cells influences microtubule dynamic instability and turnover and is associated with microtubule breakage and treadmilling.. J Cell Biol.

[40] Dixit R, Cyr R (2004). Encounters between dynamic cortical microtubules promote ordering of the cortical array through angle-dependent modifications of microtubule behavior.. Plant Cell.

[41] Chamaraux F, Ali O, Keller S, Bruckert F, Fourcade B (2008). Physical model for membrane protrusions during spreading.. Phys Biol.

[42] Dubin-Thaler B. J, Giannone G, Döbereiner H, Sheetz M. P (2004). Nanometer analysis of cell spreading on matrix-coated surfaces reveals two distinct cell states and steps.. Biophys J.

[43] Komarova Y. A, Vorobjev I. A, Borisy G. G (2002). Life cycle of MTs: persistent growth in the cell interior, asymmetric transition frequencies and effects of the cell boundary.. J Cell Sci.

[44] Busch K. E, Brunner D (2004). The microtubule plus end-tracking proteins mal3p and tip1p cooperate for cell-end targeting of interphase microtubules.. Curr Biol.

[45] Buck K. B, Zheng J. Q (2002). Growth cone turning induced by direct local modification of microtubule dynamics.. J Neurosci.

[46] Gordon-Weeks P. R (2004). Microtubules and growth cone function.. J Neurobiol.

[47] Wittmann T, Bokoch G. M, Waterman-Storer C. M (2003). Regulation of leading edge microtubule and actin dynamics downstream of Rac1.. J Cell Biol.

[48] Tomasek J. J, Hay E. D (1984). Analysis of the role of microfilaments and microtubules in acquisition of bipolarity and elongation of fibroblasts in hydrated collagen gels.. J Cell Biol.

[49] Clarke J (2009). Live imaging of development in fish embryos.. Semin Cell Dev Biol.

[50] Zhang L, Kendrick C, Jülich D, Holley S. A (2008). Cell cycle progression is required for zebrafish somite morphogenesis but not segmentation clock function.. Development.

[51] Rhodes J, Amsterdam A, Sanda T, Moreau L. A, McKenna K (2009). Emi1 maintains genomic integrity during zebrafish embryogenesis and cooperates with p53 in tumor suppression.. Mol Cell Biol.

[52] Francis F, Koulakoff A, Boucher D, Chafey P, Schaar B (1999). Doublecortin is a developmentally regulated, microtubule-associated protein expressed in migrating and differentiating neurons.. Neuron.

[53] Hu S, Chen J, Wang N (2004). Cell spreading controls balance of prestress by microtubules and extracellular matrix.. Front Biosci.

[54] Mata J, Nurse P (1997). Tea1 and the microtubular cytoskeleton are important for generating global spatial order within the fission yeast cell.. Cell.

[55] Williamson R. E, Jeon K. W, Friedlander M (1991). Orientation of cortical microtubules in interphase plant cells..

[56] Goshima G, Wollman R, Stuurman N, Scholey J. M, Vale R. D (2005). Length control of the metaphase spindle.. Curr Biol.

[57] Ciruna B, Jenny A, Lee D, Mlodzik M, Schier A. F (2006). Planar cell polarity signalling couples cell division and morphogenesis during neurulation.. Nature.

[58] Lecuit T, Lenne P. F (2007). Cell surface mechanics and the control of cell shape, tissue patterns and morphogenesis.. Nat Rev Mol Cell Biol.

[59] Harris W. A, Hartenstein V (1991). Neuronal determination without cell division in Xenopus embryos.. Neuron.

[60] Hartenstein V, Posakony J. W (1990). Sensillum development in the absence of cell division: the sensillum phenotype of the Drosophila mutant string.. Dev Biol.

[61] Pope K. L, Harris T. J. C (2008). Control of cell flattening and junctional remodeling during squamous epithelial morphogenesis in Drosophila.. Development.

[62] Jankovics F, Brunner D (2006). Transiently reorganized microtubules are essential for zippering during dorsal closure in Drosophila melanogaster.. Dev Cell.

[63] Halpain S, Dehmelt L (2006). The MAP1 family of microtubule-associated proteins.. Genome Biol.

[64] Zhang T, Zaal K. J. M, Sheridan J, Mehta A, Gundersen G. G (2009). Microtubule plus-end binding protein EB1 is necessary for muscle cell differentiation, elongation and fusion.. J Cell Sci.

[65] Straube A, Merdes A (2007). Eb3 regulates microtubule dynamics at the cell cortex and is required for myoblast elongation and fusion.. Curr Biol.

[66] Whitesides G. M, Ostuni E, Takayama S, Jiang X, Ingber D. E (2001). Soft lithography in biology and biochemistry.. Annu Rev Biomed Eng.

[67] Schneider I (1972). Cell lines derived from late embryonic stages of Drosophila melanogaster.. J Embryol Exp Morphol.

[68] Rogers S. L, Rogers G. C (2008). Culture of Drosophila S2 cells and their use for RNAi-mediated loss-of-function studies and immunofluorescence microscopy.. Nat Protoc.

[69] Riedl J, Crevenna A. H, Kessenbrock K, Yu J. H, Neukirchen D (2008). Lifeact: a versatile marker to visualise F-actin.. Nat Methods.

[70] Dogterom M, Yurke B (2008). Measurement of the force-velocity relation for growing microtubules.. Science.

