# Supplementary figures and images for: A Polarised Population of Dynamic Microtubules Mediates Homeostatic Length Control in Animal Cells
Source: PLoS Biol. 2010 Nov 16;8(11):e1000542. doi: 10.1371/journal.pbio.1000542 (PMC2982804; doi:10.1371/journal.pbio.1000542)

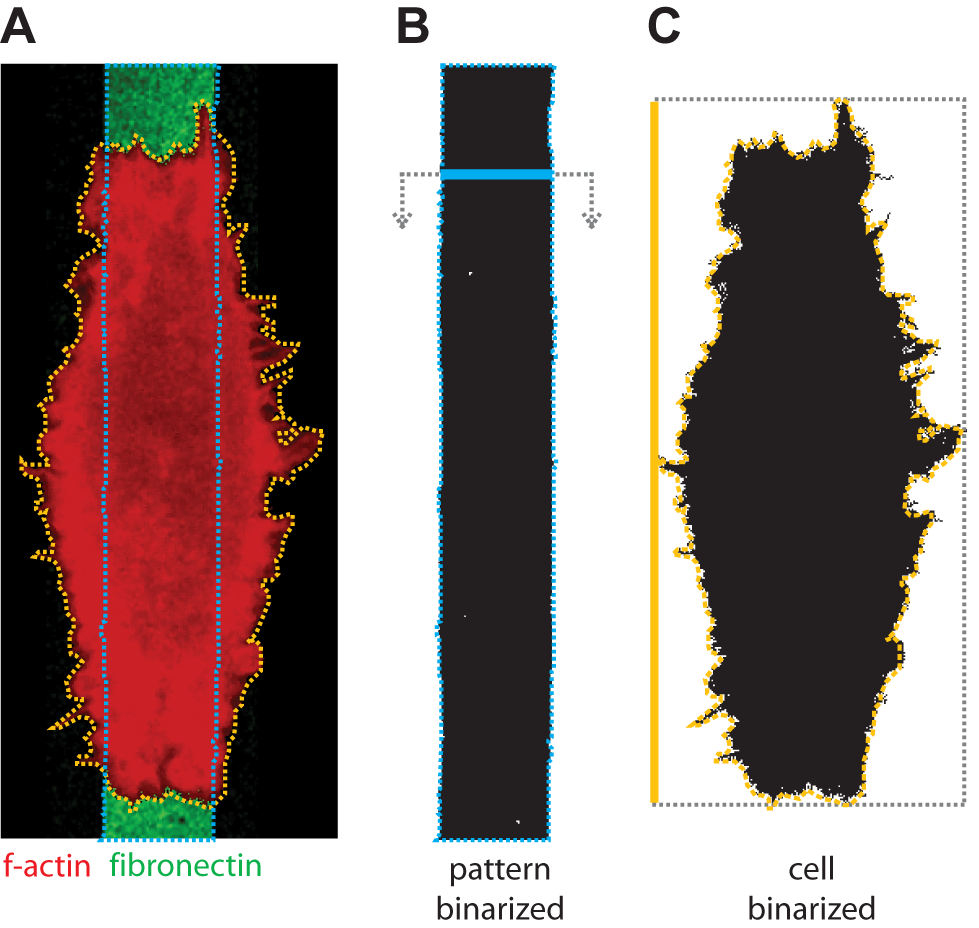

Supplement: Figure S1 — Image analysis of patterned cells. (A) Images of TRITC-labelled F-actin in cells on lines were acquired in a vertical orientation. (B) The pattern width was calculated by averaging the widths obtained at the intersection of a scanning horizontal line (blue line) with a binary pattern image (in black) as the scanning line moves vertically. (C) Lengths of patterned cells were obtained by fitting a rectangular shape to binary images of cells stained for F-actin and taking the length of the rectangle side parallel to the patterned line (yellow line). Lengths of non-patterned cells were obtained by taking the greatest distance possible between any two points along the cell boundary (Feret's diameter) (see Figure 1B). Only the length of mononuclear cells was measured. The cell ([A], yellow dotted) and the line pattern boundaries ([A], blue dotted) were obtained by thresholding image intensity. The cell and pattern boundary were successively filled inside and outside by black and white, respectively, to obtain the binary images shown in (B) and (C). Cell nucleus was segmented by the same method. (0.51 MB TIF) [file pbio.1000542.s001.tif]

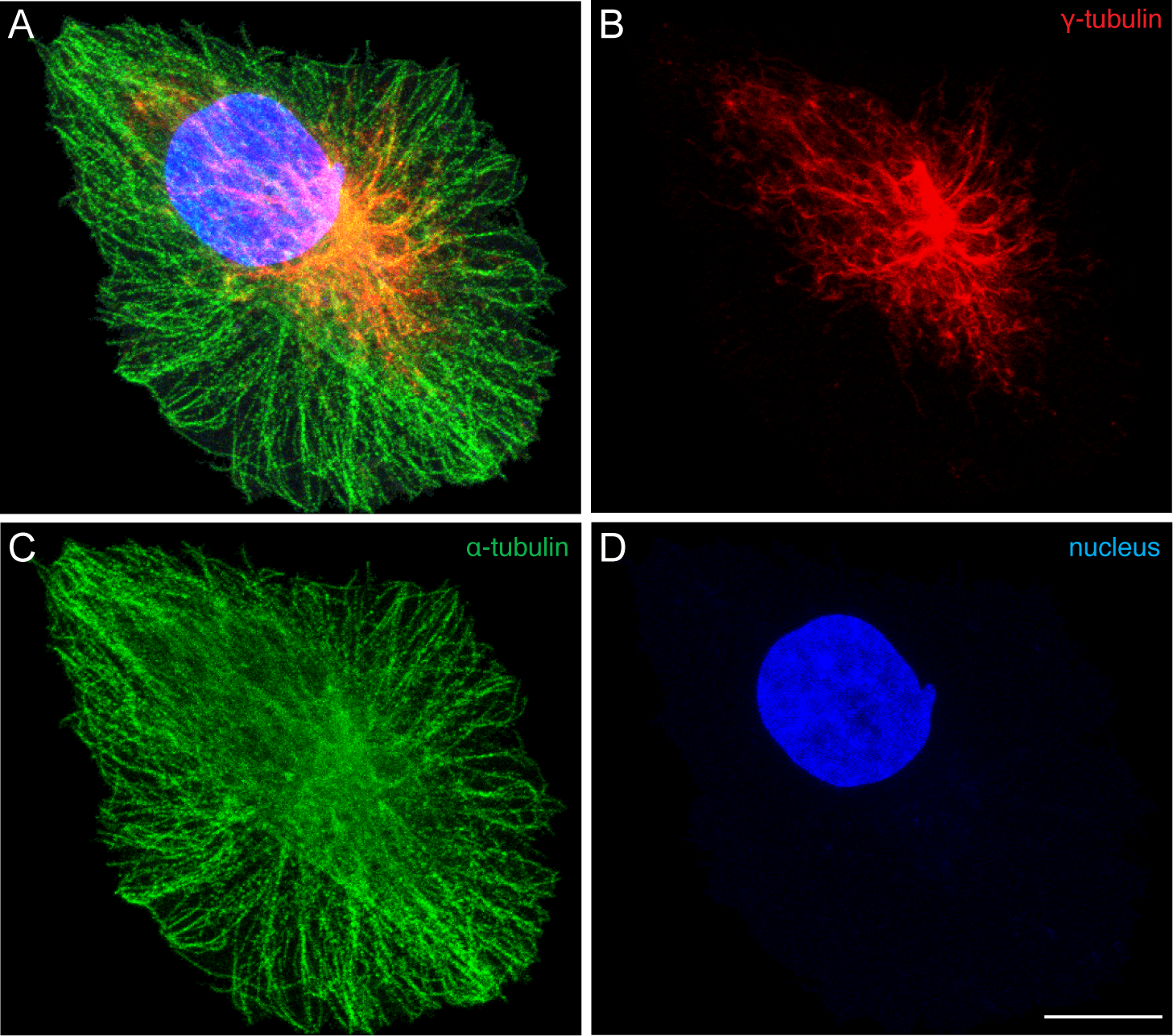

Supplement: Figure S2 — Microtubule nucleation. Microtubule nucleation sites in HeLa cells were labelled by γ-tubulin first antibody (A) and Alexa Cy5 secondary antibody (B). Microtubules were labelled by FITC-conjugated anti-α-tubulin antibody (C). Nucleus was labelled by DAPI (D). (2.53 MB TIF) [file pbio.1000542.s002.tif]

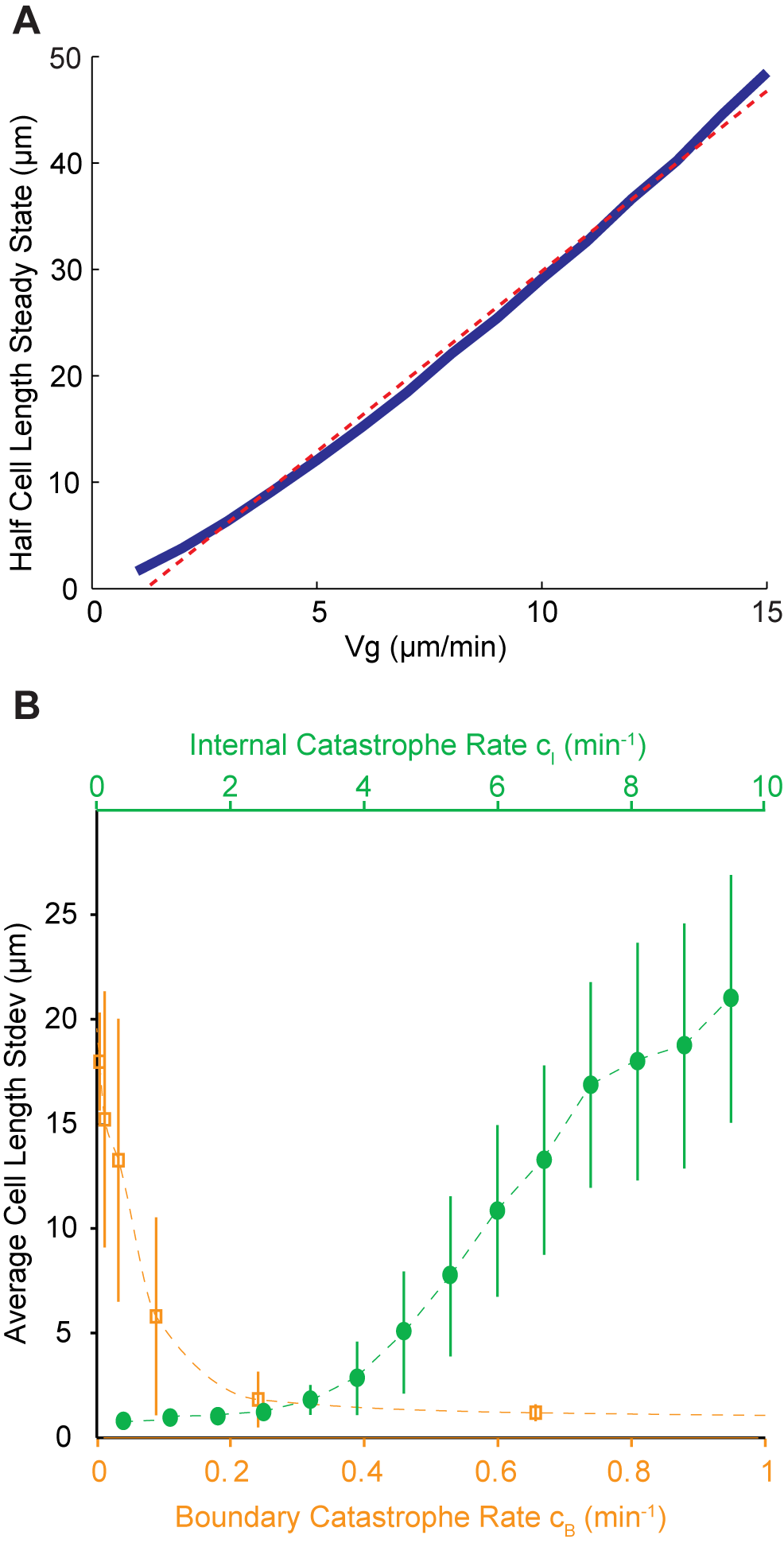

Supplement: Figure S3 — Dynamic microtubules lie along the cell bottom, close to the substrate. (A) Dynamic microtubules are concentrated within 1 µm of the substrate. Confocal zy-section projection of an HeLa cell transfected by EB3-GFP on a non-patterned fibronectin surface (B) and on a patterned fibronectin line (C). Top (D and E) and bottom (F and G) part of each HeLa cell transfected by EB3-GFP. (0.26 MB DOC) [file pbio.1000542.s003.tif]

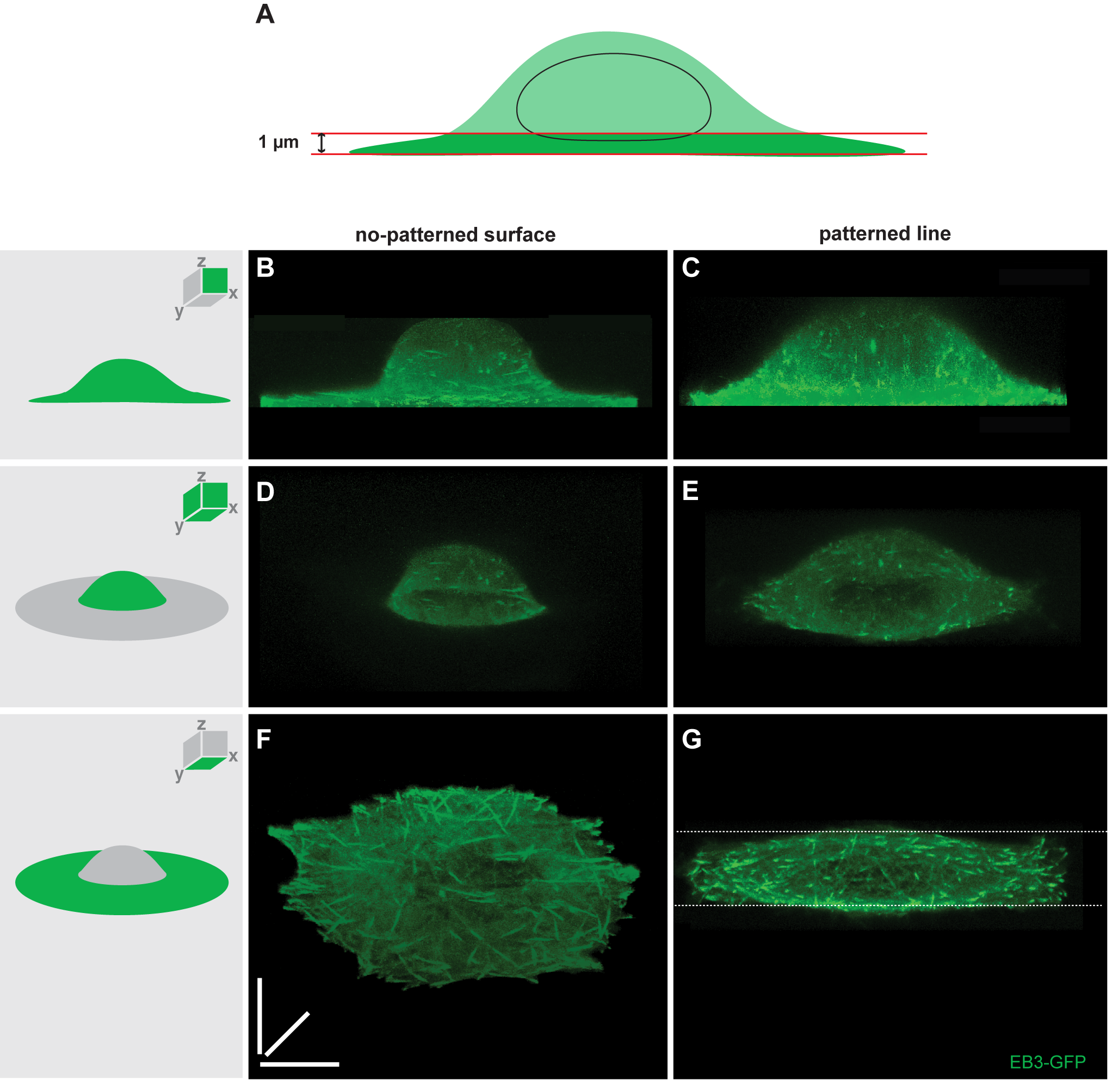

Supplement: Figure S4 — The effects of microtubule polymerisation rates on average cell length. (A) The microtubule growth rate (v g) in the model was plotted against steady-state values of cell length. The model predicts a linear relationship between microtubule polymerisation rate and cell length at steady state (y = 1.7x−2). (B) Variations in cell length (standard deviation) are shown for cells ∼44 µm long following changes in the catastrophe rates of microtubules at the cell boundary, c B (in orange), or of cytoplasmic microtubules, c I (in green). Average values were obtained by examining the variance when length was chosen to be close to experimentally determined values, using values of N m and α varying from 1 to 30 and from 0 to 30, respectively, while other parameters were kept constant (with values specified in Table 1). (2.58 MB TIF) [file pbio.1000542.s004.tif]
